# A review of interactions between peripheral and foveal vision

**DOI:** 10.1167/jov.20.12.2

**Published:** 2020-11-03

**Authors:** Emma E. M. Stewart, Matteo Valsecchi, Alexander C. Schütz

**Affiliations:** 1 Allgemeine und Biologische Psychologie, Philipps-Universität Marburg, Marburg, Germany; 2 Dipartimento di Psicologia, Universitá di Bologna, Bologna, Italy; 3 Center for Mind, Brain and Behavior, Philipps-Universität Marburg, Marburg, Germany

**Keywords:** Peripheral, foveal, review

## Abstract

Visual processing varies dramatically across the visual field. These differences start in the retina and continue all the way to the visual cortex. Despite these differences in processing, the perceptual experience of humans is remarkably stable and continuous across the visual field. Research in the last decade has shown that processing in peripheral and foveal vision is not independent, but is more directly connected than previously thought. We address three core questions on how peripheral and foveal vision interact, and review recent findings on potentially related phenomena that could provide answers to these questions. First, how is the processing of peripheral and foveal signals related during fixation? Peripheral signals seem to be processed in foveal retinotopic areas to facilitate peripheral object recognition, and foveal information seems to be extrapolated toward the periphery to generate a homogeneous representation of the environment. Second, how are peripheral and foveal signals re-calibrated? Transsaccadic changes in object features lead to a reduction in the discrepancy between peripheral and foveal appearance. Third, how is peripheral and foveal information stitched together across saccades? Peripheral and foveal signals are integrated across saccadic eye movements to average percepts and to reduce uncertainty. Together, these findings illustrate that peripheral and foveal processing are closely connected, mastering the compromise between a large peripheral visual field and high resolution at the fovea.

## Brief overview of differences between peripheral and foveal vision

Although the human eye is often compared to a photographic camera, processing across the visual field is not homogeneous like in a camera film or a digital sensor. First, there are gaps in sensory information due to several anatomical properties of the eye: (a) there are no photoreceptors in the optic disc, where the axons of the retinal ganglion cells exit the eyeball: this leads to a blind spot (Mariotte, 1740, cited after Ferree & Rand, 1912; Grzybowski & Aydin, 2007). (b) The center of the retina contains only cone, but no rod photoreceptors (Schultze, 1866; Oesterberg, 1935; Curcio, Sloan, Kalina, & Hendrickson, 1990), leading to a central scotoma under dark illumination conditions. (c) Because photoreceptors are located on the back side of the retina, away from the light, blood vessels cast shadows on them (Purkinje, 1819; von Helmholtz, 1867; Evans, 1927; Adams & Horton, 2002). The second striking difference to a photographic camera is that the processing of visual signals varies quite dramatically across the visual field. Here, an important distinction arises between the center of the visual field, called the fovea, and the rest, called the periphery.^1^ We only briefly highlight some of the key differences in processing and perception between the fovea and the periphery because these have been reviewed in detail elsewhere (Strasburger, Rentschler, & Juttner, 2011; Whitney & Levi, 2011; Rosenholtz, 2016; Simpson, 2017; Knotts, Odegaard, Lau, & Rosenthal, 2018), and because we want to focus on their interactions in the later sections.

The inherent differences between the fovea and the periphery can already be observed in the anatomy of the retina. Cone photoreceptor density peaks in the fovea, and declines toward the periphery (Oesterberg, 1935; Curcio et al., 1990). In the subsequent processing of visual signals, more receptors converge on a single retinal ganglion cell in the periphery than in the fovea (Curcio & Allen, 1990), where even one-to-one connections of receptors to ganglion cells occur. This leads to an over-representation of the fovea that is continued throughout the visual hierarchy in the lateral geniculate nucleus (Malpeli & Baker, 1975) and visual cortex (e.g. Holmes, 1918; Daniel & Whitteridge, 1961; Horton & Hoyt, 1991; Dumoulin & Wandell, 2008), as well as in visuomotor structures like the superior colliculus (Robinson, 1972; Ottes, Gisbergen, & Eggermont, 1986; Chen, Hoffmann, Distler, & Hafed, 2019).

Unsurprisingly, this architecture of the visual system has severe consequences for vision. Basic visual performance measures, such as acuity (Aubert & Foerster, 1857; Wertheim, 1894; see Anstis, 1974, for a beautiful and influential demonstration) and contrast sensitivity (e.g. Robson & Graham, 1981; Pointer & Hess, 1989) peak at the fovea and decline toward the periphery.^2^ Spatial summation, on the other hand, increases toward the periphery (Ricco, 1877; Sloan, 1961; Wilson, 1970). Finally, peripheral vision is also subject to larger uncertainty in the localization of features and objects (Rentschler & Treutwein, 1985; Levi & Klein, 1986). This is impressively illustrated by models that produce spatially distorted images that cannot be distinguished from their original, undistorted images in the periphery (Balas, Nakano, & Rosenholtz, 2009; Freeman & Simoncelli, 2011; Koenderink, Valsecchi, van Doorn, Wagemans, & Gegenfurtner, 2017). Beyond these low-level effects, the peripheral visual field is also more heavily affected by crowding (Korte, 1923; Bouma, 1970; for reviews see Levi, 2008; Pelli, 2008; Pelli & Tillman, 2008; Whitney & Levi, 2011; Strasburger, 2020). Crowding occurs when the recognition of one object is impaired by the presence of other objects in the vicinity. The critical distance between the objects that is necessary for identification increases toward the periphery.

Overall, a picture emerges where peripheral vision is not merely a blurry version of foveal vision, but lacks details about individual objects and their shapes and position (e.g. Aubert & Foerster, 1857; Lettvin, 1976). This can be described as a more texture-like perception, where access to individual elements is limited, while summary statistics are still available (for reviews see Strasburger et al., 2011; Rosenholtz, 2016; Whitney & Yamanashi Leib, 2018; Strasburger, 2020).

**Figure 1.**
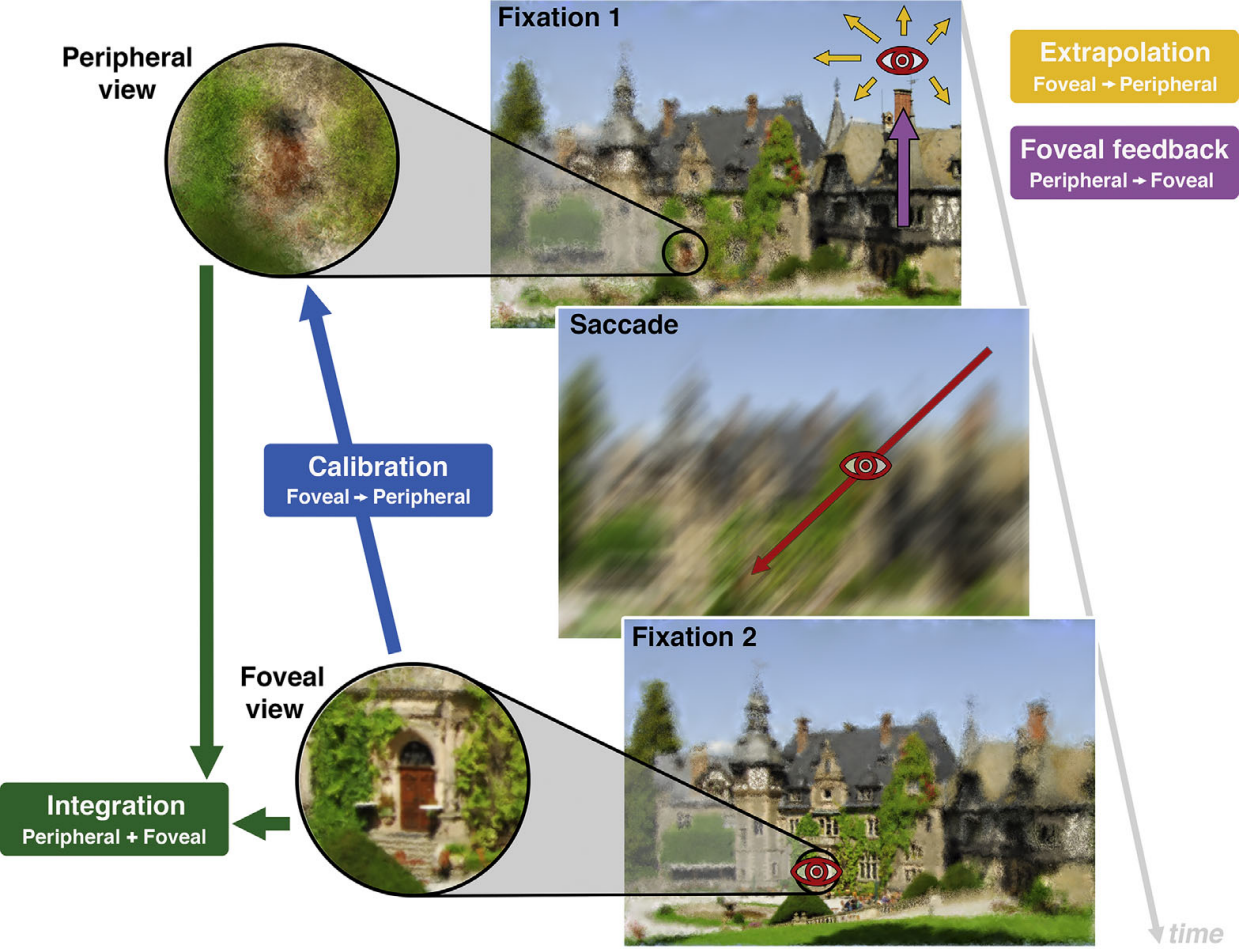
Schematic illustration of interactions between peripheral and foveal vision. The images illustrate differences between peripheral and foveal vision and the typical sequence of transsaccadic vision. Yellow, purple, green, and blue arrows indicate the direction of information flow. During fixation, peripheral vision is characterized by uncertainty in position (illustrated by spatial disarray using the Eidolon Factory (Koenderink et al., 2017); the degradation is overemphasised for the purpose of illustration), reduced spatial resolution, and increased crowding. Foveal appearance is extrapolated towards the periphery (yellow arrows; Extrapolation of foveal information to the periphery) and peripheral object recognition is supported by foveal feedback processing (purple arrow; Foveal feedback signals supporting peripheral object recognition). A saccadic eye movement (red arrow) brings an object of interest (here the castle door) to the fovea. During the saccade, vision is impaired (Dodge, 1900; for reviews see Matin, 1974; Volkmann, 1986; Wurtz, 2008; Binda & Morrone, 2018) by several factors, such as motion blur (Burr & Ross, 1982; but see Castet & Masson, 2000), masking due to the clear pre- and postsaccadic image (Matin, Clymer, & Matin, 1972; Campbell & Wurtz, 1978; Duyck et al., 2016), and active reduction of contrast sensitivity (e.g. Volkmann, 1962; Burr, Morrone, Ross, 1994; Diamond, Ross, Morrone, 2000). Differences between peripheral and foveal appearance might be compensated by transsaccadic re-calibration (blue arrow; Re-calibration of peripheral and foveal vision). Information from successive fixations might be stitched together by transsaccadic integration (green arrows; Transsaccadic integration of peripheral and foveal information).

There is a consensus that foveal and peripheral vision accomplish two opposing goals with limited processing resources: foveal vision allows for maximal acuity and contrast sensitivity in a small region around the gaze position, whereas peripheral vision allows for a large field of view, albeit with lower resolution, contrast sensitivity, higher positional uncertainty, and more crowding. Despite these opposing goals, and the large differences in processing, foveal and peripheral vision are not clearly separated in phenomenology. This suggests that peripheral and foveal vision are closely intertwined, despite their obvious differences. In the following sections, we will discuss three core questions about the interaction of peripheral and foveal vision (Figure 1). (1) How is the processing of peripheral and foveal signals related during fixation? (2) How are peripheral and foveal signals re-calibrated? (3) How is peripheral and foveal information stitched together across saccades? Notice that we concentrate here on the interactions between peripheral and foveal vision within the boundaries of feature perception and object recognition. Beyond the scope of this review are studies on gaze control, which show that peripheral and foveal vision both contribute to determining the “when and where” of gaze shifts (e.g. Laubrock, Cajar, & Engbert, 2013; Ludwig, Davies, & Eckstein, 2014; Nuthmann, 2014; Nuthmann & Malcolm, 2016; Tatler, Brockmole, & Carpenter, 2017).

## Foveal-peripheral interactions during fixation

The aforementioned literature has described clear differences between foveal and peripheral vision on both a physiological level and a perceptual level. This raises the question as to whether foveal and peripheral vision are working independently, or whether there are direct interactions between the two. In this section, we review some physiological and perceptual findings showing that foveal and peripheral vision are tightly interconnected, even in the absence of eye movements. These interactions might be geared to enhance peripheral vision, thereby making peripheral and foveal vision more homogeneous.

### Foveal feedback signals supporting peripheral object recognition

**Figure 2.**
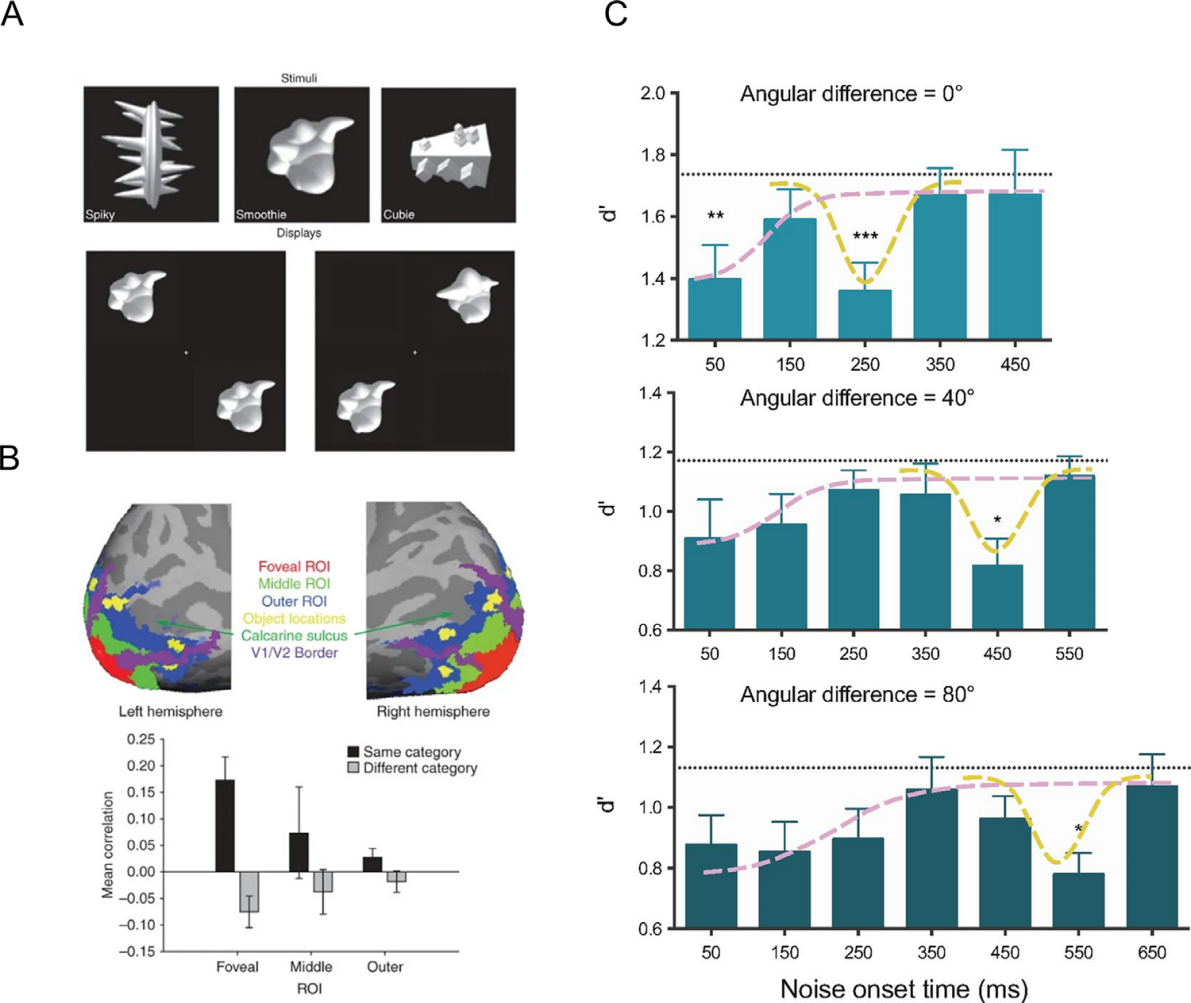
Peripheral object discrimination and foveal feedback. (A) Experimental paradigm and stimuli in the original paradigm (Williams et al., 2008). Participants had to categorize objects from three different categories. In each trial, two objects from the same or a different category were shown in the peripheral visual field and participants had to judge whether the two objects came from the same or a different category. Figure modified with permission from Williams et al. (2008). (B) Results in brain imaging (Williams et al., 2008). Region of interest (ROI) for further analysis and average correlations of brain activity elicited by objects of the same or a different category. Same-category correlations are higher than chance only in the foveal ROI, but not in peripheral ROIs outside of stimulus presentation. Figure modified with permission from Williams et al. (2008). (C) Behavioural consequences and time course of the effect (Fan et al., 2016). Presenting a noise mask in the fovea impairs peripheral object categorisation. The subpanels show conditions that require different amounts of mental rotation of the objects. The time course of the effect is modulated by the necessary amount of mental rotation. The pink dashed line indicates early detrimental effects of noise that are presumably related to the distraction of attention (Beck & Lavie, 2005). The yellow dashed line indicates the detrimental effect of noise that is related to the interference with foveal-feedback signals. Figure modified with permission from Fan et al. (2016).

A rather recent finding in the history of vision science concerns the processing of peripherally displayed stimuli in foveal retinotopic cortex. The discovery of this effect is particularly interesting because it was observed originally in brain imaging, and its behavioural consequences were only reported later. In their seminal study, Williams, Baker, de Beeck, Shim, Dang, Triantafyllou, & Kanwisher (2008) provided the first evidence for the crucial role of foveal processing for the perception of objects in the periphery. They presented objects from three different categories in the peripheral visual field, and recorded BOLD activity in functional magnetic resonance imaging (fMRI) while observers performed a perceptual comparison task on those objects. Strikingly, it was possible to decode information about the object category from BOLD activity in foveal-retinotopic cortex, although the objects were presented in the peripheral visual field. Traditionally, one would expect relevant brain activity only in the peripheral-retinotopic cortex (i.e. at the projections of the locations where the objects were presented). These decoded signals were specific to foveal-retinotopic cortex and were not present at nonstimulated peripheral locations (Figures 2A,B). Interestingly, the decoding of object category was only possible when observers performed the object comparison task, but not when they performed a color comparison task. The authors argued that the decoding in foveal-retinotopic cortex relied on feedback signals that originate in higher-tier visual areas.

Several years later, a series of studies established the crucial role of these foveal feedback signals for the perception of objects in the periphery. Disrupting foveal processing with transcranial magnetic stimulation (TMS) leads to an impairment of peripheral object discrimination (Chambers, Allen, Maizey, & Williams, 2013). This was the first causal evidence that foveal feedback signals are indeed beneficial for peripheral object discrimination. A similar impairment of peripheral object discrimination can be accomplished by presenting an incongruent distractor object (Weldon, Rich, Woolgar, & Williams, 2016), or visual noise in the fovea (Fan, Wang, Shao, Kersten, & He, 2016). As a positive consequence, peripheral object discrimination can be facilitated by the presentation of a congruent object in the fovea, even when this foveal object is rendered invisible by a subsequent pattern mask (Yu & Shim, 2016).^3^

The timing of the interference effect is a key property for its understanding. Both TMS and foveal stimuli are only effective if they occur well after the peripheral object, which is further evidence for feedback signals. Fan et al. (2016) identified two distinct effects of foveal noise (Figure 2C): an early effect at short delays when the presentation of the peripheral objects and the noise are temporally overlapping, and, after a short recovery period, a late effect. The early effect is presumably related to the distraction of attention away from the peripheral objects (Beck & Lavie, 2005) because it occurs only when the locations of the peripheral objects are not predictable and because it does not depend on the type of discrimination task. The late effect is presumably related to the interference with the foveal feedback signals because it occurs only for an object recognition task requiring fine spatial details, but not for an object recognition task with blurred objects, or a speed discrimination task with moving stimuli. Interestingly, the exact time course of the effects depends critically on the processing speed for the peripheral object. When the processing of the peripheral object is impaired by adding a secondary task, or by requiring mental rotation of the object, the effective window of foveal distractors is delayed. As shown in the classic studies by Shepard and colleagues (Shepard & Metzler, 1971; Cooper & Shepard, 1973), the duration of mental rotation is a linear function of the necessary rotation angle. Consistently, the detrimental effect of foveal noise is delayed according to the amount of mental rotation that is necessary (Fan et al., 2016). In line with the spatial specificity of the decoding in the fMRI study (Williams et al., 2008), it has been shown that peripheral distractors are not effective in the late time window when foveal distractors are effective (Weldon et al., 2016).

The specificity of the interference effect is another key property. The role of foveal feedback seems to be graded depending on the complexity of the peripheral stimulus and the perceptual discrimination task. Peripheral orientation discrimination of gratings is less modulated by delayed foveal stimuli than peripheral object discrimination (Yu & Shim, 2016). Similarly, shape discrimination is more heavily impaired by delayed foveal distraction than color discrimination (Weldon, Woolgar, Rich, & Williams, 2020). As mentioned above, object recognition for blurred objects is not impaired (Fan et al., 2016), suggesting that foveal feedback is only relevant for, or involved in, the analysis of fine spatial details. Object categorization itself is only impaired by foveal distraction on a subordinate level (duck versus non-duck) and a basic level (bird versus non-bird), but not on superordinate level (animal versus non-animal; Ramezani, Kheradpisheh, Thorpe, & Ghodrati, 2019). Overall, these results illustrate that peripheral processing is only sufficient for coarse analysis and recognition, and that foveal processing is necessary for the analysis of object details.

These findings on the crucial role of foveal feedback for peripheral object categorization can be linked to several other empirical findings and theories on visual perception. The special role of feedback processing for perception has been emphasized in the literature multiple times. Lamme and Roelfsema (2000) proposed that feedback processing is necessary for attentive grouping of object features and conscious perception. From this perspective, the interruption of foveal feedback should be particularly harmful for categorization tasks requiring the integration of multiple features. In their reverse hierarchy theory, Hochstein and Ahissar (2002) proposed that feedforward signals from lower to higher areas are responsible only for “vision at a glance.” This fast route provides information about superordinate and basic level object categorization, but lacks details about subordinate object categorization and individual features. Feedback signals from higher to lower areas are necessary for “vision with scrutiny.” This slower route provides information about low level features, such as color, orientation, shape, etc. The graded effect of foveal feedback (Fan et al., 2016; Yu & Shim, 2016; Ramezani et al., 2019; Weldon et al., 2020) is consistent with these assumptions of the reverse hierarchy theory.

Furthermore, there are some more specific findings on the differences between foveal and peripheral vision that can be linked with foveal feedback signals. Several fMRI studies have shown that the representation of different stimulus categories is not homogeneous across the visual field, but that there are clear eccentricity biases: faces and words show a foveal bias, and buildings show a peripheral bias in their cortical representation (Levy, Hasson, Avidan, Hendler, & Malach, 2001; Hasson, Levy, Behrmann, Hendler, & Malach, 2002; Malach, Levy, & Hasson, 2002). These eccentricity biases suggest a functional specialization of the foveal and peripheral visual fields that might be related to the role of foveal feedback. A more important role of feedback signals for foveal than for peripheral vision has also been proposed by Zhaoping (2019), based on differences in the processing of disparity in the fovea and the periphery (Zhaoping, 2017; Zhaoping & Ackermann, 2018).

### Extrapolation of foveal information to the periphery

As mentioned in the introduction, the large differences between foveal and peripheral processing are not reflected in conscious perception, which is remarkably homogeneous across the visual field (Chong & Treisman, 2003; for review see Cohen, Dennett, & Kanwisher, 2016; and see Haun, Tononi, Koch, & Tsuchiya, 2017 for an alternative view). How this homogeneity of visual experience comes about is still debated intensely. One possible interpretation is that perception in the periphery merely appears sharper (Galvin, O'Shea, Squire, & Govan, 1997) and more detailed than it actually is, a process called *inflation* (Solovey, Graney, & Lau, 2015; Knotts et al., 2018; Odegaard et al., 2018). This could originate from imprecise and biased metacognitive decision making (Odegaard, Chang, Lau, & Cheung, 2018; but see Abid, 2019), leading to the impression of knowing more about the peripheral visual field than is actually the case. Consistent with this view, several studies have shown systematic overconfidence for highly uncertain stimuli, for instance due to inattention (Rahnev, Maniscalco, Graves, Huang, de Lange, & Lau, 2011), or due to missing sensory information in the blind spot (Ehinger, Häusser, Ossandón, & König, 2017) and the foveal scotoma during scotopic viewing (Gloriani & Schütz, 2019). Another possible interpretation is that information is actually transferred or extrapolated from the fovea to the periphery. This *extrapolation* could be regarded as equivalent to perceptual (and possibly neural) filling-in of gaps in the visual field (for reviews see Pessoa, Thompson, & Noë, 1998; Komatsu, 2006; de Weerd, 2006), such as the blind spot (Fick & Du Bois-Reymond, 1853; Volkmann, 1853; Ramachandran, 1992; Durgin, Tripathy, & Levi, 1995) or the foveal scotoma during scotopic viewing (Linden, 1997; Gloriani & Schütz, 2019). A related phenomenon might also be the perceptual filling-in of surface features within boundaries, for instance the water color illusion (Pinna, Brelstaff, & Spillmann, 2001, Pinna, Werner, & Spillmann, 2003), or neon color spreading (Bressan, Mingolla, Spillmann, & Watanabe, 1997). Here, we concentrate on the case of extrapolation, because it is more directly relevant with respect to foveal-peripheral interactions than inflation.

In the following paragraphs, we will describe evidence for extrapolation coming from three different experimental paradigms: peripheral brightness estimation, peripheral feature binding errors, and the uniformity illusion; and finally, will discuss the limitations of extrapolation. A rather simple and straightforward piece of evidence for extrapolation has been reported in a study about brightness estimation. When observers are asked to estimate the brightness of a peripheral target area without looking at it, their estimations are affected by the brightness at the fovea (Toscani, Gegenfurtner, & Valsecchi, 2017). Most notably, this effect is only present when the peripheral area and the foveal area belong to the same object. This rules out the explanation that this effect is a simple response bias, and indicates that foveal information about brightness is indeed extrapolated toward the periphery, but only within the boundaries of objects.

As mentioned in the introduction, the binding of features for individual objects in a crowd is impaired in the periphery. Wu, Kanai, and Shimojo (2004) showed in a seminal study that this lack of detailed object information leads to misbinding errors in the periphery based on information from the center. In their illusion, observers saw a cloud of dots with two motion directions (up- and downward) and two colors (red and green). When motion direction and color are associated differently in the centre (e.g. downward + green) than in the periphery (upward + green), observers show a strong bias to perceive the same feature binding in the periphery as in the center. Later studies showed that this effect occurs quite rapidly (Kanai, Wu, Verstraten, & Shimojo, 2006) and generalizes to other feature combinations, such as color and orientation (Suzuki, Wolfe, Horowitz, & Noguchi, 2013), and motion and orientation (Stepien & Shevell, 2015). The effect is also quite robust, as it is not necessary to have exactly the same colors in the center and the periphery (Wang & Shevell, 2014; Shevell & Wang, 2016). However, a critical precondition is a perfect correlation of features in the centre (e.g. all horizontal bars are red, and all vertical bars are green): misbinding in the periphery does not occur if this correlation is broken by keeping one feature constant (e.g. only horizontal bars) or by randomly combining features (Suzuki et al., 2013).

**Figure 3.**
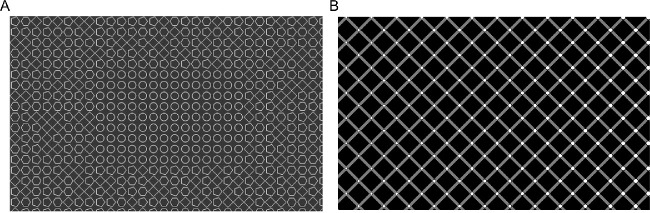
Visual illusions cancelling or inducing differences between foveal and peripheral appearance. (A) Uniformity illusion. Texture statistics are different in the central (only circles) and peripheral (squares, penta- and hexagons) part of the stimulus. After maintaining fixation on the centre for a few seconds, the peripheral part of the stimulus appears identical to the centre. Figure modified with permission from Otten, Pinto, Paffen, Seth, & Kanai (2017). (B) Extinction illusion. Each crossing of the grey bands contains a small white disk, but these disks are only visible in the fovea. Figure modified with permission from Ninio and Stevens (2000).

Extrapolation of foveal information to the periphery can also be observed in the uniformity illusion (Otten et al., 2017; see also Kanai, 2005). This illusion is based on a textured pattern with different statistics in the center and the periphery (Figure 3A). After maintaining fixation on the center for a while, the whole pattern appears to be uniformly structured following the statistics in the center. The illusion is effective for a remarkably long list of visual features, such as shape, orientation, luminance, motion, etc. The uniformity illusion might be considered as an inverse effect of fading and filling-in of textures (e.g. Bergen, 1991; Gyoba, 1997; Stürzel & Spillmann, 2001; Hindi Attar, Hamburger, Rosenholtz, Götzl, & Spillmann, 2007), where a differentially textured area blends in with the surround texture after a while. Although the pattern in the uniformity illusion appears uniform, some information about the actual stimulus properties in the periphery is preserved. Suárez-Pinilla, Seth, and Roseboom (2018) investigated the representation of peripheral information by measuring the tilt aftereffect. The tilt aftereffect (Gibson & Radner, 1937) describes the perceptual phenomenon that after adaptation to a certain tilt (say vertical), the perceived tilt of following stimuli is slightly biased away from the adapted tilt. Because the tilt aftereffect can be localized in retinotopic coordinates (Knapen, Rolfs, Wexler, & Cavanagh, 2010; Mathôt & Theeuwes, 2013; Nakashima & Sugita, 2017; Zimmermann, Weidner, & Fink, 2017), it can be used to measure which orientation is represented in the periphery: the actual orientation of stimuli in the periphery, or the perceived orientation that stems from the fovea. The results (Suárez-Pinilla et al., 2018) showed that the tilt aftereffect in the uniformity illusion depends on the actual stimulus orientation. This suggests that the stimulus orientation is still represented in early visual cortex, even if the whole pattern appears uniform at the foveal orientation. This is an interesting dissociation from illusory contours (e.g. Kanizsa, 1979) and perceptually invisible patterns, which can both generate a tilt aftereffect (Smith & Over, 1975; He & MacLeod, 2001), and suggests that the uniformity illusion arises at a higher level of processing.

Although the uniformity illusion occurs for a large list of visual features, it is not a general phenomenon. There are several illusions that show persistent differences between foveal and peripheral appearance, despite physically uniform patterns. The most well-known example might be the Hermann grid (Hermann, 1870; Lingelbach, Block, Hatzky, & Reisinger, 1985; Spillmann, 1994; Geier, Bernáth, Hudák, & Séra, 2008), in which illusory dark spots appear at the crossings of white bands only in the periphery, but not in the fovea. With each new fixation, the dark spots disappear at the fixated location and reappear at the previously fixated location in the periphery. Other examples are the extinction illusion (Ninio & Stevens, 2000), where bright disks at the crossings in a Hermann grid disappear only in the periphery (Figure 3B), and the honeycomb illusion (Bertamini, Herzog, & Bruno, 2016, Bertamini, Cretenoud, & Herzog, 2019), where small barbs attached to the crossings in a hexagonal texture are only perceived in the fovea, but not in the periphery. Those features follow gaze position and seem to jump with every eye movement. Most importantly, the disks in the extinction illusion and the barbs in the honeycomb illusion can be perceived in the periphery if they are shown without the square or hexagonal texture in the background (Bertamini et al., 2019). This means that the invisibility of the small elements is not merely a consequence of lower peripheral resolution. Irrespective of how these illusions come about, they vividly illustrate the limits of extrapolation in matching peripheral and foveal appearance. This indicates that extrapolation itself is not sufficient to explain the absence of differences in foveal and peripheral appearance. In the next section, we will discuss transsaccadic re-calibration as a potential mechanism to match appearance in peripheral and foveal vision.

### Open questions

Foveal feedback signals and extrapolation are both rather recent empirical findings and therefore our knowledge about those effects is still scarce, leaving many open questions for further investigation. Here, we want to highlight some of the more fundamental questions.

1. *What is the functional goal of foveal feedback signals?* Although there is converging evidence that foveal feedback signals are clearly beneficial for the recognition of objects in the periphery (e.g. Fan et al., 2016; Weldon et al., 2016), the functional goal of this feedback mechanism is still ambiguous. One possibility might be that the foveal feedback signals reflect the specialization and division of labor between foveal and peripheral processing. As mentioned above, there is a foveal eccentricity bias for some object categories, like faces or text, and a peripheral eccentricity bias for other categories, like houses or places (Levy et al., 2001; Hasson et al., 2002; Malach et al., 2002). Hence, the foveal feedback effect might be only one leg of a connection between peripheral and foveal processing that is actually bidirectional. In this case, one would expect an inverse, peripheral feedback effect for object categories that show a peripheral eccentricity bias, such as houses. This remains to be tested, because all studies on the foveal feedback effect used objects that exhibit a foveal eccentricity bias. Another possibility might be that the foveal feedback signals reflect the usage of generally superior capabilities of foveal processing. From this view, peripheral vision is upgraded by further processing in foveal vision, and one would expect that the foveal feedback effect is a unidirectional effect from peripheral to foveal vision only. In particular, this might be related to the typical exploration sequence where objects are detected in peripheral vision first, before they are brought to foveal vision by saccadic eye movements (see sections below).
2. *Are foveal-feedback signals only relevant for the instantaneous recognition of objects or also for learning to recognize objects?* Humans can recognize objects with ease at an amazing speed (Thorpe, Fize, & Marlot, 1996), both in the fovea and the periphery (Thorpe, Gegenfurtner, Fabre-Thorpe, & Bülthoff, 2001). Despite advances in the analysis of imaging data and the modeling of object recognition (for reviews see Kriegeskorte, Mur, & Bandettini, 2008; Kriegeskorte, 2015), it is still unclear how humans achieve this amazing ability (for reviews see Peissig & Tarr, 2007; Kourtzi & Connor, 2011; Gauthier & Tarr, 2016), and even more so how they learn to recognize objects in the course of development. Future studies will need to assess whether foveal feedback signals are not just facilitating the instantaneous recognition of peripheral objects, but also the learning of unfamiliar objects.
3. *Is peripheral object recognition affected by foveal scotomata?* A loss of vision in the fovea can occur due to different retinal diseases, for instance, in age-related macular degeneration (for a review see Jager, Mieler, & Miller, 2008). It is still a matter of debate whether the compensation of pathological scotomata is supported by a reorganization of visual cortex (for reviews see Wandell & Smirnakis, 2009; Dumoulin & Knapen, 2018). Nevertheless, if foveal feedback processing is necessary for peripheral object recognition, impairments of foveal vision might also affect the recognition of objects in the intact peripheral visual field.
4. *Why is there extrapolation from the fovea to the periphery in some visual textures, but not in others?* Visual illusions vividly illustrate that foveal appearance is extrapolated to the periphery in some textures, such as the uniformity illusion (see Figure 3A), but not in others, such as the Hermann grid or the extinction illusion (see Figure 3B). At present, it is unclear which factors limit extrapolation in the latter cases. Conceptually, one could think of the process of extrapolation as a competition between (extrapolated) signals from the fovea and weak sensory signals at the periphery. In that case, one would expect stronger extrapolation when the foveal signals are repetitive and indicative of a uniform texture, and when the peripheral signals are more uncertain.

## Re-calibration of peripheral and foveal vision

As we have outlined in the introduction, peripheral and foveal vision differ in many aspects, and, for the most part, peripheral vision achieves the ability to represent a large portion of the visual field by giving up the ability to represent all the elements that it might contain individually, and with high acuity. But how does our visual system deal with the problem of keeping together the representations it builds, using sensory machineries that differ so dramatically?

Of course, one solution to the problem would be to ignore those anisotropies. Our visual system could use peripheral and foveal vision for qualitatively different purposes, such as locomotion, navigation, attentional guidance in the periphery, and object recognition in the fovea, which could make the binding of foveal and peripheral representations unnecessary. However, from a phenomenological point of view, this does not seem to be what is happening. We, as humans, do not have the impression that our eyes move to places for reasons that we cannot explain, and that things appear to us in our fovea out of nowhere. We have the impression that when we move our eyes the world is stable and unchanging, and that we actually perceive a relatively uniform world in front of us: yet the way we sense the world changes with every saccade. Besides the mechanisms of inflation and extrapolation that we described in the previous section, this could be due to the fact that that foveal and peripheral vision are, to a certain extent, calibrated (i.e. our visual system represents perceptual dimensions sensed through foveal and peripheral vision in a way that at least partially discounts the differences between the respective sensory processes).

One of the factors that might help us to associate foveal and peripheral appearance into a stable world representation is transsaccadic learning. Specifically, transsaccadic learning can establish the rules for matching central and peripheral sensory input (i.e. calibrate foveal and peripheral vision) and change them, if needed, once they are established (i.e. re-calibration). The idea that transsaccadic learning subtends our ability to match foveal and peripheral appearance is not new, and was already expressed very clearly by Hermann von Helmholtz in his Treatise on Physiological Optics (Helmholtz, 1867; Quote from the English translation, 1925):

Now when we perceive any object in indirect vision, and thus have received a limited impression of it on a peripheral part of the retina, and then turn the eye so as to look straight at it, we get afterwards an impression of the same object with the same apparent size on the center of the retina; and thus we can gradually learn by experience when a certain peripheral impression is the same in quality and size as a central impression.

As far as its accuracy extends, this renders it possible to learn to judge of objects by their form and apparent size even in indirect vision (Helmholtz & Southall, 1925, p. 186).

More recently, the idea that learned sensorimotor contingencies are what allow us to bind sensory information acquired through different sensory modalities, such as foveal and peripheral vision, has been one of the main tenets of the sensorimotor account of vision and visual consciousness by O'Regan and Noë (2001). In particular, they clearly predicted that a repeated transsaccadic change would have the effect of unifying the two physically different stimuli, sensed foveally and peripherally, into a common phenomenological experience, for instance, in the case of color appearance:

Using a device to measure eye movements connected to a computer, it should be possible to arrange stimulation on a display screen so that whenever an observer looks directly at a patch of colour it appears red, but whenever the observer's eye looks away from the patch, its color changes to green. The rather counterintuitive prediction from this is that, after training in this situation, the observer should come to have the impression that green patches in peripheral vision and red patches in central vision are the same color (O'Regan & Noë, 2001, p. 952).

This suggestion is at the core of the paradigms that have uncovered the role of transsaccadic learning in associating foveal and peripheral vision. First, however, one related field of research on sensorimotor learning needs to be discussed, because some of the evidence from these experiments might allow us to better characterize the phenomenon of transsaccadic perceptual re-calibration. This field of study concerns the possibility of gaze-contingent biases in color perception.

### Gaze-contingent color perception

The first experiments on gaze-contingent color perception were conducted by Kohler (1951). He asked one observer to wear bipartite colored spectacles, blue on the left and yellow on the right side, for two months. From a retinocentric perspective on color vision, one would expect that the observer should adapt to the filtering properties of the spectacles as long as they fixate on one location. Every time they move their eyes horizontally, they should, however, experience a dramatic change in the color percept in the portion of the retinocentric visual field that moves between filtering areas. In addition, they should remain aware of the left-right anisotropy in color filtering because they would experience color in the portion of the cranio-centric visual field surrounding the spectacles’ blue-yellow boundary every time they move their eyes. This was initially the case for Kohler's observer, but after 10 days of wearing the spectacles, they reported a strongly decreased color percept associated with eye movements. Interestingly, they still saw changes in colors when they moved their head while keeping their eyes fixed, which points to a possible limitation in our visual system's ability to integrate visuomotor contingencies in our experience of the world.

Most of the findings by Kohler (1951) were replicated by Leppmann & Wieland (1966), including the fact that at the end of the trial, after a night's sleep and without wearing the glasses, the observer reported seeing a cranio-centric afterimage when moving their eyes: in other words, an afterimage that would not move across the room when they moved their eyes left and right while keeping their head still. Interestingly, the afterimage disappeared when the chair that the observer was sitting on was turned, which likely produced a vestibulo-ocular reflex that compensated for head rotation. Other studies, however, seemingly failed to replicate the original study. Harrington (1965) had observers wear split glasses, for up to 146 days. His observers did not report that they became unaware of the filtering properties of the glasses over time, and when gaze-angle dependent changes in color perception were measured (by means of achromatic adjustments and color picking), no noteworthy effects were found. McCollough (1965) also failed to measure gaze-dependent changes in color appearance after wearing bipartite colored spectacles, and suggested that some of the observations by Kohler (1951) were possibly due to the fact that changing gaze orientation also changed the portion of the visual stimulus that fell in the adapted peripheral visual field. Notice that the peripheral visual field is likely to be adapted independent of gaze orientation, because observers mostly prefer to gaze forward in craniotopic coordinates. Having the stimulus extend into the adapted peripheral visual field might have in turn changed its foveal appearance due to long-range contrast effects.

**Figure 4.**
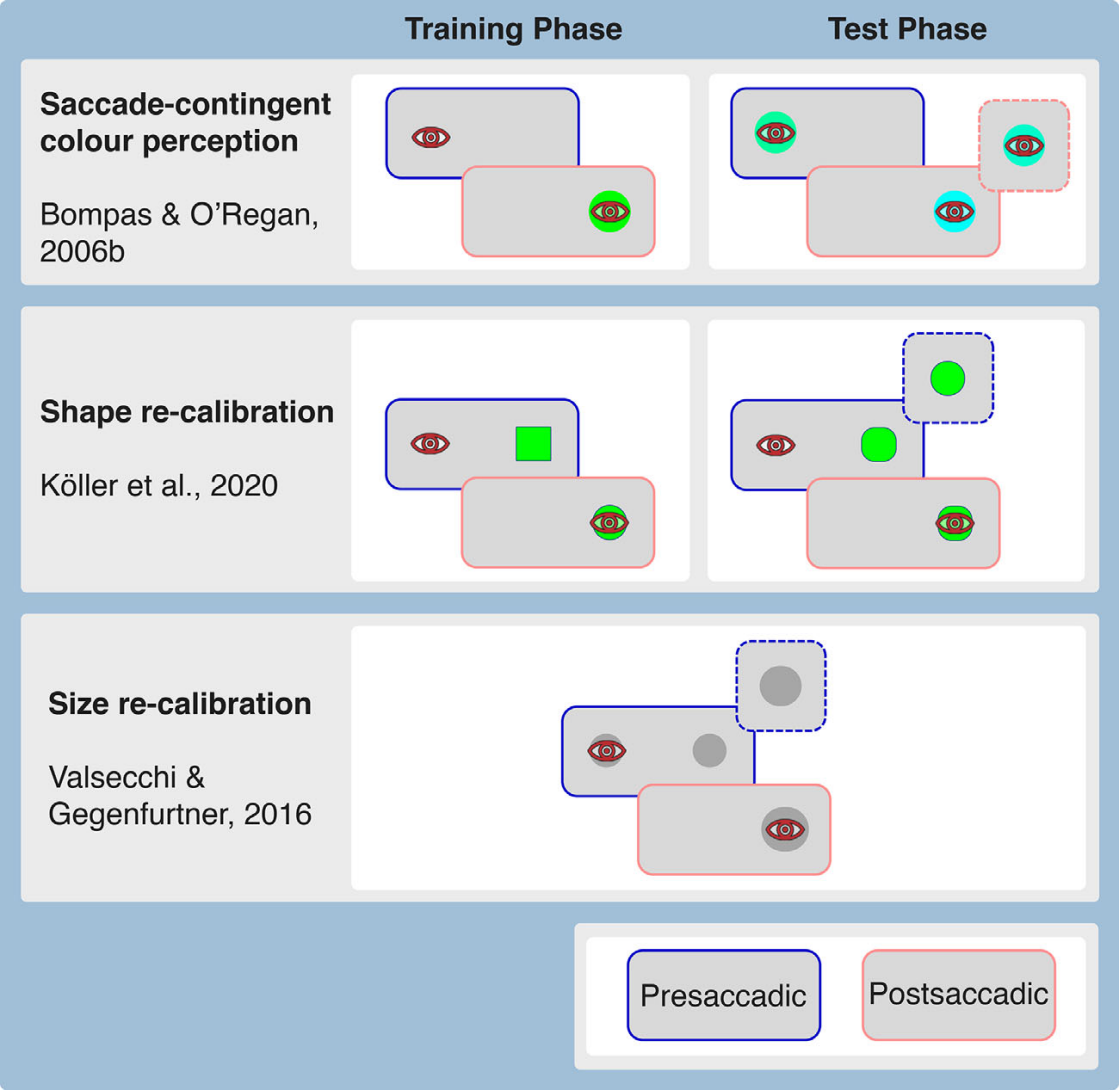
Relevant aspects of the main paradigms used in the literature to test the effects of sensorimotor learning on perception. The joined screens represent the pre- and postsaccadic physical situation (blue and light red contours, respectively). The smaller screens surrounded by dashed lines represent the supposed appearance after learning. Top Row: saccade-contingent colour perception. Notice that, in this case, the observer never sees any peripheral stimuli, and saccade direction alters postsaccadic foveal appearance. In the test phase the perceptual effect is assessed by having the observer compare the colour of the pre- and postsaccadic (foveal) stimuli. Center Row: shape re-calibration. Notice that, in this case, we only represent the case of a swapped object, which turns from a square into a circle transsaccadically in the training phase. After training the peripheral stimulus appears more circular. In the test phase, the perceptual effect is assessed by having the observer compare the shape of the presaccadic (peripheral) stimulus with the shape of the postsaccadic (foveal) stimulus. Bottom Row: size re-calibration. In this case, the observer experiences a transsaccadic increase in size, which increases the perceived size of the peripheral stimulus. Notice that in this paradigm there are no distinct training and test phases. Within the same trial, size perception is assessed first by having the observer compare two stimuli that are presented simultaneously. In the second part of the trial, the observer executes the saccade and experiences the transsaccadic change.

Whether color adaptation contingent on eye position is a true phenomenon or an artifact, we would suggest that the specific prediction of the sensorimotor account (O'Regan & Noë, 2001) is that perturbations of color vision should be intimately related to eye movements, both temporally, and possibly in terms of the specific type of eye movement that brings about the eye-in-orbit position change (e.g. a saccade as compared to vestibulo-ocular reflex). This was more directly tested in two studies by Bompas and O'Regan, (2004) and Bompas and O'Regan (2006). In the first study, they found that as little as four hours of adaptation with bipartite colored spectacles could produce a shift in perceived color measured transsaccadically. For instance, they had observers execute a saccade first to the left and then to the right, and presented a patch at fixation when the eyes landed. This produced a reliable, albeit relatively small shift in perceived color opposite to the color of the spectacles (i.e. a form of color adaptation contingent on saccade direction). Similar results were observed in the second study (Bompas & O'Regan, 2006; replicated by Richters & Eskew, 2009), where instead of having observers wear colored spectacles, they presented stimuli at the gaze landing position, whose color (red or green) was contingent on the direction of the saccade (Figure 4, top row). In this case, 40 minutes of training already produced a measurable shift in perceived color that was opposite to the color polarity associated with a given saccade direction.

All in all, the evidence seems to suggest that, while color perception contingent on gaze position is an uncertain phenomenon, which might take days to develop, associating stimuli to saccade directions produces changes in perception in a remarkably short time frame. Associating color perception to saccade directions is not a form of foveal-peripheral re-calibration, because what is changed is not the association between the peripheral stimulation and the corresponding foveal stimulation across saccades, but the appearance of a stimulus in the foveal visual field, conditioned on how the visual field came to be centered at a given location. Yet, it suggests again that sensorimotor contingencies can change visual perception, pointing toward the possibility of transsaccadic re-calibration. Furthermore, the results point to two aspects that might be relevant for the research on transsaccadic re-calibration. First, there seems to be a discrepancy between the very fast development of effects contingent on saccade direction, and the more sluggish adaptation to colored spectacles that was reported in the older experiments. However, this might simply be due to the fact that the emergence of subjectively relevant effects, that would be reported by observers, requires more thorough training and stronger changes in perceived color. In any case, this opens the possibility that qualitatively different forms of sensorimotor learning could develop both over a few trials, and over days of uninterrupted training. Second, the results seem to indicate that eye and head movements are not necessarily equivalent when it comes to establishing sensorimotor contingencies, although in daily life humans are constantly orienting gaze toward peripheral locations using combined head and eye-in-head movements (e.g. Einhäuser, Schumann, Bardins, Bartl, Böning, Schneider, & König, 2007).

### Transsaccadic re-calibration

As we anticipated, proper demonstrations of transsaccadic perceptual re-calibration of peripheral and foveal appearance would need to use a paradigm similar to the armchair experiment suggested by O'Regan and Noë (2001). That paradigm differs from the ones that were devised to demonstrate gaze or saccade-contingent color perception, because it involved not simply associating the appearance of a given stimulus to a specific saccadic direction, but transsaccadically associating the peripheral and foveal appearance of a stimulus. Over the last decade and a half, a growing body of studies have been conducted that effectively demonstrated perceptual re-calibration through transsaccadic learning. They can be roughly organized along two lines: those that investigated transsaccadic re-calibration contingent on specific objects, and those that investigated transsaccadic re-calibration using a more general mapping scheme.

The study that established the main elements of the paradigms that have been subsequently used to investigate object-specific transsaccadic learning is the work of Cox, Meier, Oertelt, and DiCarlo (2005). They repeatedly presented complex “greeble” stimuli in the periphery (Gauthier & Tarr, 1997) and had observers saccade toward them. In the “normal” condition, no transsaccadic change took place, but in the “swapped” conditions the greeble systematically changed identity before the saccade landed. Their paradigm took advantage of the fact that our ability to detect changes during saccades is largely reduced (e.g. McConkie & Currie, 1996), so that observers tend to be unaware of the manipulation they are experiencing in transsaccadic-learning paradigms. They observed that when tested after exposure to as few as 120 swaps, observers became less likely to report a difference between the presaccadic peripheral stimulus and the postsaccadic foveal one, as compared to their performance with “normal” stimuli. The authors interpreted this as evidence that the observers were recognizing objects contingent on appearance but also retinal position. A subsequent study by Li and DiCarlo (2008) found that the IT neurons of monkeys trained in a similar paradigm start to respond to nonpreferred objects that are associated transsaccadically with preferred ones at “swapped” locations.

Although the study by Cox and colleagues (2005) showed that observers can learn very specific transsaccadic associations (i.e. a combination of saccade direction, object identity, presaccadic peripheral appearance, and postsaccadic foveal appearance), subsequent studies tested the possibility that more general transsaccadic perceptual mappings could be learned (see Figure 4, middle row, for a typical example). Herwig and Schneider (2014) had observers perform saccades toward circular and triangular stimuli filled by a sinusoidal grating. One of the stimuli was swapped during the saccade (i.e. the stimulus’ pattern changed consistently to a lower or higher spatial frequency), whereas the other stimulus stayed unchanged. After the training phase, observers were tested by having to compare the spatial frequency of a presaccadic target with the spatial frequency of a probe stimulus presented foveally after the saccade. The results indicated that, selectively for swapped objects, the observers’ judgments were consistently biased toward the postsaccadic spatial frequency that they had experienced in the training phase. In a different experiment where the effects of training were tested by using a simple visual search task, observers tended to saccade toward a stimulus when its expected postsaccadic spatial frequency, rather than its physical peripheral spatial frequency, matched the one of the instructed search target.

The association between peripheral and foveal appearance for spatial frequency can be established despite additional transsaccadic changes to spatial location and even object shape. For example, the tendency to look at the stimulus whose predicted postsaccadic spatial frequency matches the instructed target in visual search can be established even if the swapped stimulus also changes shape from a circle to a triangle during the saccade in the learning phase (Weiß, Schneider, & Herwig, 2014). Subsequent results from the same research group extended these results. Herwig, Weiß, and Schneider (2015) demonstrated that transsaccadic associations can also be established when a geometric property, such as shape, changes transsaccadically, and swapped and normal stimuli are identified by a surface property such as colour. Köller, Poth, and Herwig (2020) showed that the effects of transsaccadic learning tend to saturate when the shape change reaches a certain magnitude, but they still occur. Learning that a square becomes a circle when fixated still makes the peripheral square look more roundish. Paeye, Collins, Cavanagh, and Herwig (2018) showed that peripheral and foveal appearance of shape can be learned even if the stimulus appears first in the periphery and then in the fovea and no saccade occurs, and even if a temporal gap of up to one second intervenes between peripheral and foveal stimulus. This is consistent with the results of Valsecchi and Gegenfurtner (2016), who showed that re-calibration can be established without eye movements when the stimulus moves slowly on the screen toward the fovea. Herwig, Weiß, and Schneider (2018) returned to using spatial frequency as a learned stimulus dimension and horizontal saccades for training, but varied the spatial configuration of saccadic starting and landing point, and position of the stimulus that had to be judged. They found that judgments in the testing phase were maximally influenced by learning when the testing occurred under the exact same conditions as during training (i.e. saccades were horizontal and the stimulus to be memorized was at the saccade goal). Offsetting the probe stimulus above or below the horizontal midline reduced the learning effect, regardless of whether, during testing, the saccade was made to the usual location or to the probe location.

Taken together, the results by the various experiments using the “normal” versus “swapped” presentation showed that observers can learn to associate a change in a given visual dimension (spatial frequency and shape) to a specific object and the execution of a saccade, and that this association biases their perceptual judgments when comparing presaccadic peripheral and postsaccadic foveal stimuli. This strongly suggests that our transsaccadic experience shapes the way our peripheral and central visual fields appear to us. Two aspects of these studies, however, somewhat limit their potential to reveal the mechanisms that subtend the observed learning effect and the temporal dynamics of its acquisition. First and most importantly, their paradigms invariably probe the memorised representation of the peripheral stimulus, so it is still possible that rather than modifying the way our visual field appears to us, transsaccadic learning only changes the way stimuli are remembered across saccades. Second, these studies usually involve a separate training and test phase, which limits their use for evaluating how transsaccadic re-calibration develops across trials. As anticipated, a second category of studies moved away from the “normal” versus “swapped” presentation paradigm to overcome these shortcomings.

The first study in this category was performed by Bosco, Lappe, and Fattori (2015), who had observers perform saccades to peripheral rectangular stimuli. One of the edges of the stimulus was systematically extended or contracted during the saccade, which produced a larger or smaller stimulus, as well as a shift in the stimulus’ center of mass. They observed that repeated exposure to this manipulation produced both a change in the size of the saccades, i.e. saccade adaptation, and a change in the perceived size of the peripheral stimulus. Crucially, they used a manual reproduction task and a verbal estimation task, without saccades, to probe the perceived size of the stimulus, ensuring that the change in reported size is not contingent on comparing a foveal stimulus with the transsaccadic memory trace of a peripheral stimulus. The second study was performed by Valsecchi and Gegenfurtner (2016). They also investigated the transsaccadic learning of size changes. In order to avoid comparing central and peripheral appearance through memory, they used a direct comparison between foveal and peripheral stimulus before the saccade (see Figure 4, bottom row). Because observers compare two stimuli that are simultaneously viewed, for an indefinite amount of time, this method of probing size perception should minimize the role of memory and tap directly into the appearance of foveally and peripherally viewed stimuli. Given that the perceptual judgment and the saccade task were coupled in a single trial, their paradigm also allowed for the evaluation of transsaccadic re-calibration as it was being acquired. Additional experiments in the study suggested that our visual system preferentially uses the prediction from periphery to fovea for the purpose of re-calibration, and that transsaccadic feedback at one location re-calibrates appearance at the mirror location in the opposite hemifield.

Valsecchi and colleagues, in their contribution to this Special Issue (Valsecchi, Cassanello, Herwig, Rolfs, & Gegenfurtner 2020), took advantage of the potential temporal resolution of this method to investigate the relationship between saccade adaptation and perceptual re-calibration. There are at least two lines of evidence suggesting that these two phenomena are closely related. Saccade adaptation has been shown to alter the geometric properties of the perceptual visual field, which in turn can modify the perceived shape of objects (Garaas & Pomplun, 2011), and there is very recent evidence that saccade adaptation per se can modify the perceived size of peripheral stimuli (Pressigout, Paeye, & Doré-Mazars, 2020).^4^ Moreover, when saccade adaptation and perceptual re-calibration are established within the same paradigm, their strength tends to correlate across observers (Bosco et al., 2015), although transsaccadic re-calibration of perceived size can be established in the absence of saccade adaptation if the expansion or contraction of the target stimulus is orthogonal to the saccade vector (Bosco, Rifai, Wahl, Fattori, & Lappe, 2020). Specifically, Valsecchi and colleagues (2020) investigated the temporal and spatial properties of transsaccadic size re-calibration. They found that in response to transsaccadic changes oscillating in time across trials, perceptual judgments oscillate as well, with temporal dynamics similar to those that can be observed when saccade adaptation is measured in response to intrasaccadic steps that oscillate in time (Cassanello, Ohl, & Rolfs, 2016; Cassanello, Ostendorf, & Rolfs, 2019). They also confirmed that transsaccadic re-calibration acquired at one location generalises to the mirror location, whereas saccadic adaptation does not. This seems to indicate that these two forms of transsaccadic learning might rely on a common prediction error signal, but that their implementation is qualitatively different.

Although all the studies that we have described confirm that transsaccadic perceptual changes can re-calibrate foveal and central vision, one might still question whether results obtained with such an artificial paradigm, requiring the physical change of the stimulus contingent on an eye movement, might generalize to more natural conditions. Recently, Eymond, Seidel Malkinson, and Naccache (2020) showed that the effects of the Ebbinghaus illusion are opposite in peripheral vision (i.e. the perceived size of the central stimulus of the display becomes attracted towards the size of the inducers) compared with the canonical situation in foveal vision (where larger inducers make the central stimulus appear smaller and vice versa). Crucially, repeatedly looking at the Ebbinghaus display over time abolished the reverse illusion. This provides proof that perceptual discrepancies between central and peripheral vision that are not induced by transsaccadic manipulations can also be re-calibrated through transsaccadic learning.

### Open questions

In general, the studies on gaze-contingent color perception and those on transsaccadic re-calibration have shown that visuomotor experience can help us to accommodate some of the sensory differences that exist between peripheral and foveal vision. While the studies so far shed light on many aspects of transsaccadic re-calibration, there are still many open questions that need to be addressed.

1. *Does appearance really change?* Due to saccadic suppression, observers are largely unaware of the transsaccadic manipulations that are applied by experimenters in re-calibration paradigms (see Figure 4, center and bottom rows). This has been taken as an indication that observers are not changing their judgments strategically to conform to the experimental demands (e.g. Herwig & Schneider, 2014; Valsecchi & Gegenfurtner, 2016). Nonetheless, it is possible that the observers get explicit feedback from the postsaccadic stimulus, and use it to change their perceptual decision criterion afterward. There is one finding that speaks against it: both observers who detect the transsaccadic change, and observers who do not detect it, show equivalent levels of re-calibration (Köller et al., 2020). One would expect that the observers who attribute their prediction error to a trick played on them by the experimenter would show a reduced re-calibration effect, if the latter depends on them noticing the transsaccadic prediction error and attributing it to a perceptual mis-judgment. At the same time, however, nondetectors might be such because they are less able to produce a precise transsaccadic prediction and thus experience a weaker transsaccadic prediction error, which in turn would produce a weaker re-calibration. Two opposing mechanisms might contribute to give the impression that nondetectors and detectors are identical in terms of re-calibration. One alternative way of dealing with the question of whether strategic aspects are involved in transsaccadic re-calibration would be to investigate whether, and how, transsaccadic learning changes the stimulus representation in retinotopic visual areas with fMRI or magnetoencephalography (MEG). This would also help to answer the question of whether transsaccadic learning changes the representation in the periphery, in the fovea, or both. One could assume that the peripheral representation, being less precise, will be the first to change, but foveal representations can change as well (Bompas & O'Regan, 2006).
2. *What is the relationship between saccade-contingent perception and transsaccadic re-calibration?* In a saccade-contingent perception paradigm (see Figure 4, top row) observers learn to associate a certain saccadic eye movement to the appearance of a certain foveal stimulus, as evidenced by the fact that their perceptual judgments seem to be partially dictated by the statistics of the stimuli they experience (Bompas & O'Regan, 2004; Bompas & O'Regan, 2006). In a Bayesian framework, one could consider the re-calibrated appearance of a peripheral stimulus as the posterior obtained by combining a likelihood (the peripheral sensory signal) with a prior (the learned statistics of the foveal stimulus), which is contingent on the identity of the peripheral stimulus and possibly also visual field location or saccade direction. It is an open question as to what extent the effects observed in transsaccadic re-calibration studies are not due to re-calibration, but due to associations contingent on presaccadic identity and location. Those associations could be learned independently of the specific transsaccadic or peripheral-foveal correspondence of the feature that is being re-calibrated. One way of evaluating this would be to expose observers to foveal and peripheral stimuli with different respective statistical properties (e.g. larger size, greener colour, higher spatial frequency, etc.) in separate blocks, and then check whether this exposure changes judgments in perceptual comparisons between the fovea and periphery.
3. *Can re-calibration be associated with movements other than saccades?* While in our daily life most of the time an object will move from the visual periphery to the fovea because a saccade was made toward it, there are also other scenarios where this may occur. For instance, an object might get closer to the fovea because the object that is being pursued moved closer to it, or an object might land on the fovea because a combined eye and head movement was executed. There are both reasons for and against the notion that pursuit eye movements could also produce perceptual re-calibration. On the one hand, re-calibration has been observed even without any eye movements (Valsecchi & Gegenfurtner, 2016; Paeye et al., 2018), which suggests that any time an object moves toward the fovea, re-calibration is established, including in the case of pursuit. On the other hand, when we pursue a target, attention is allocated near the target itself (e.g. van Donkelaar & Drew, 2002; Lovejoy, Fowler, & Krauzlis, 2009; Chen, Valsecchi, & Gegenfurtner, 2017; for review see Schütz, Braun, & Gegenfurtner, 2011), meaning that the stimulus approaching the target will be largely unattended, which might prevent re-calibration, assuming that the lack of attention is the reason why re-calibration is not established when transsaccadic changes are applied to the stimulus that gaze is being averted from (reverse change experiment in Valsecchi & Gegenfurtner, 2016). As for combined eye-head gaze shifts, it is an open question as to whether re-calibration would be established, and whether it would be attached to the whole gaze-shift or to the eye movement component alone.
4. *Spatial specificity?* One of the unsolved questions in the field of transsaccadic re-calibration concerns its spatial specificity. Herwig and colleagues (2018) found that re-calibration established with horizontal (leftward and rightward) saccades did not generalize to oblique saccades. However, Valsecchi and Gegenfurtner (2016, confirmed by Valsecchi et al., 2020) found that re-calibration acquired at one specific location (left or right) generalised to the opposite hemifield. Multiple differences between the two experimental paradigms (discussed in Valsecchi et al. 2020) might explain this discrepancy, and a systematic test of all potentially relevant parameters is needed.
5. *Different phenomena at different time scales?* On the one hand, the earlier studies on gaze-contingent perception reported that the effects took days to develop (Kohler, 1951; Leppmann & Wieland, 1966). Independent of whether the effects they reported are replicable, it is a common experience that getting fully used to wearing a new pair of prescription glasses can take hours, if not days. On the other hand, transsaccadic perceptual re-calibration can be established within a time frame of a few minutes, and some effects might be evident after very few exposures to transsaccadic change (Valsecchi et al., 2020). One open question for the future is whether learning over multiple days simply results from the accumulation of learning over the single exposures, or whether qualitatively different processes are responsible for the consolidation of learning over longer time frames.

## Transsaccadic integration of peripheral and foveal information

**Figure 5.**
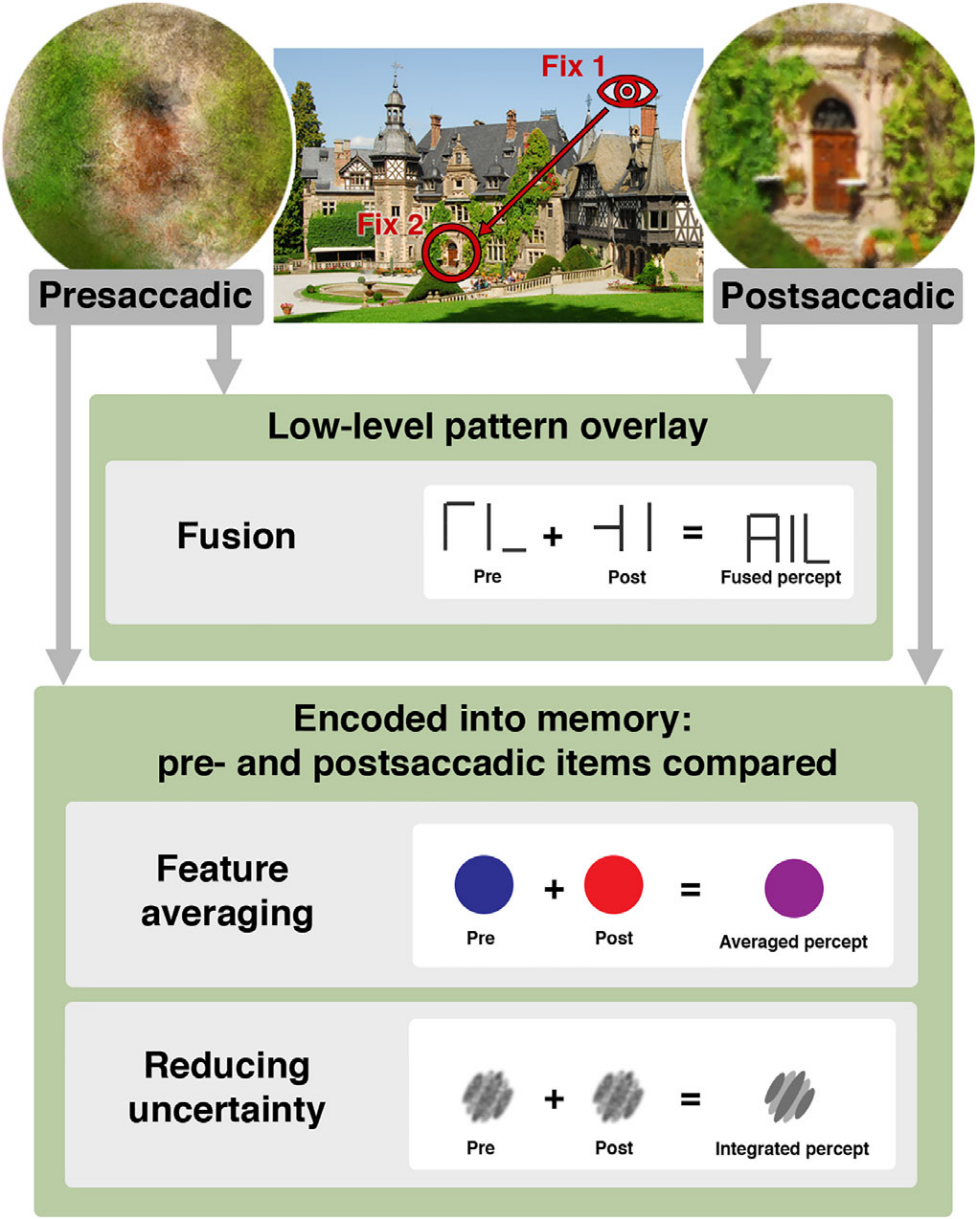
Overview of differing mechanisms of transsaccadic stimulus comparison and combination, from low-level pattern overlay (fusion) to high-level feature averaging and uncertainty reduction. When a saccade is made to a stimulus (this figure shows an example of a saccade being made to the door of Rauischholzhausen Castle), the presaccadic, peripheral percept of the stimulus must be reconciled with the postsaccadic, foveal percept of the stimulus. At the lowest level, this can occur as an overlay of pre- and postsaccadic patterns (fusion). Note that fusion only occurs under specific conditions: a task that does not require precise spatial alignment, and a weak foveal stimulus (Fusion: low level pattern overlay). On a higher level of feature combination, the pre- and postsaccadic stimuli are encoded into visual working memory, and then the pre- and postsaccadic stimulus feature information can be combined (Integration of feature information across saccades). The ultimate percept of the stimulus may be a result of both pre- and postsaccadic stimulus features (feature averaging); furthermore, the transsaccadic percept may be more reliable than either the pre- or postsaccadic percept alone, as a result of transsaccadic integration (reducing uncertainty).

The execution of a saccadic eye movement leads to a large discontinuity in visual processing and the visual system must reconcile the presaccadic, peripheral view of an object or location, with its postsaccadic, foveal counterpart. One of the mechanisms that may be used to counteract this problem could be via re-calibration; however, it has also been proposed that pre- and postsaccadic information are combined, or integrated, to alleviate the differences between peripheral and foveal vision. Many such combinative processes have been conflated under the sweeping term of “transsaccadic integration,” but we will focus on those studies that specifically address the question of how peripheral presaccadic and foveal postsaccadic information are combined, rather than the larger literature on retention and combination of information across saccades in general. The nature of this transsaccadic memory resource has been widely studied and debated, and it seems likely that transsaccadic information transfer may be reliant on both visual working memory (VWM; Baddeley & Hitch, 1974; Irwin, Zacks, & Brown, 1990), and a pre-attentive iconic memory mechanism (Sperling, 1960; Germeys, Graef, Eccelpoel, Verfaillie, 2010; for a recent comprehensive review on transsaccadic memory, see Aagten-Murphy & Bays, 2018; see also Irwin, 1996, and Higgins & Rayner, 2014, for further reviews). Unless stated otherwise, in the following section, the terms “pre- and postsaccadic” refer to stimuli that are viewed first in the periphery, and then foveated after the saccade; as such, we focus on those studies that examine the integration of pre- and postsaccadic stimuli that appear in the same spatiotopic (world-centred rather than eye-centred) location.^5^ This section covers cases where pre- and postsaccadic information are combined, from the lowest level pattern overlay, to higher-level feature averaging and uncertainty reduction (Figure 5). It further discusses the extents and limitations of transsaccadic integration.

### Fusion: Low level pattern overlay

The question of how successively presented stimuli are combined is not a new one and was initially posed in terms of temporal integration during fixation, with early results showing that such stimuli can be integrated to form a composite image (Hogben & di Lollo, 1974). The explanation for this phenomenon was that these stimuli are held in a short-term visual store, or iconic memory, and fused together (di Lollo, 1977). When the question arose as to how discontinuous information may be combined into a coherent percept across saccades, temporal integration seemed like an obvious mechanism, and it was posited that, as was observed during fixation, pre- and postsaccadic stimuli should be overlayed and fused to form a composite image (Jonides, Irwin, & Yantis, 1982). Initially, this seemed like a plausible explanation, with results from the transsaccadic measures mimicking those of temporal integration studies during fixation. However, this transsaccadic effect found by Jonides et al. (1982) was later found to have arisen from methodological problems caused by the persistence of phosphors on the screen (Jonides, Irwin, & Yantis, 1983; Rayner & Pollatsek, 1983), and the conclusion was that a low-level overlay of transsaccadic information was not possible. Subsequent studies argued against any form of transsaccadic fusion existing: for example, Irwin et al. (1990) found that direct fusion of pre- and postsaccadic stimuli does not occur, and Bridgeman & Mayer (1983) found that observers could not fuse an array of pre- and postsaccadic dots to determine which dot was missing. Similarly, O'Regan & Lévy-Schoen (1983) found no evidence of fusion of lines presented in the same spatiotopic coordinates across saccades. These results seemed to suggest rather conclusively that such low-level transsaccadic feature overlay was not possible, and that the temporal integration mechanism during fixation could not be extrapolated to account for perceptual continuity across saccades. This led to the suggestion that perceptual stability is achieved through a more general memory mechanism, rather than a specific “buffer” that retains precise spatiotopic details across saccades (Irwin, 1991).

Recently, however, it has been demonstrated that under very specific circumstances, fusion can occur. Paeye, Collins, and Cavanagh (2017) showed that a presaccadic stimulus (vertical bar) could be overlayed with a postsaccadic stimulus (horizontal bars) to form a composite image of vertical and horizontal components, but the effect was only observed when the postsaccadic stimulus had a reduced contrast, and when the fusion task did not require a precise spatial alignment of stimuli (this result was replicated in a TMS study, using the same stimulus parameters: Edwards, VanRullen, & Cavanagh, 2017).

### Integration of feature information across saccades

This section covers three broad themes of transsaccadic integration: how peripheral information can directly alter the postsaccadic percept, how the combination of peripheral and foveal information can result in a weighted average, and how the utilisation of both peripheral and foveal information is beneficial to perception. Unlike the aforementioned low-level pattern overlay described by fusion, the following sections describe cases where stimulus information is extracted from the presaccadic stimulus, retained in visual working memory, and then compared or combined with the postsaccadic stimulus. This represents a higher level of transsaccadic feature integration and does not produce a mere composite of the pre- and postsaccadic stimuli, but can produce both a weighted average of, and benefit from, the combination of peripheral and foveal information.

#### Presaccadic information alters postsaccadic perception

**Figure 6.**
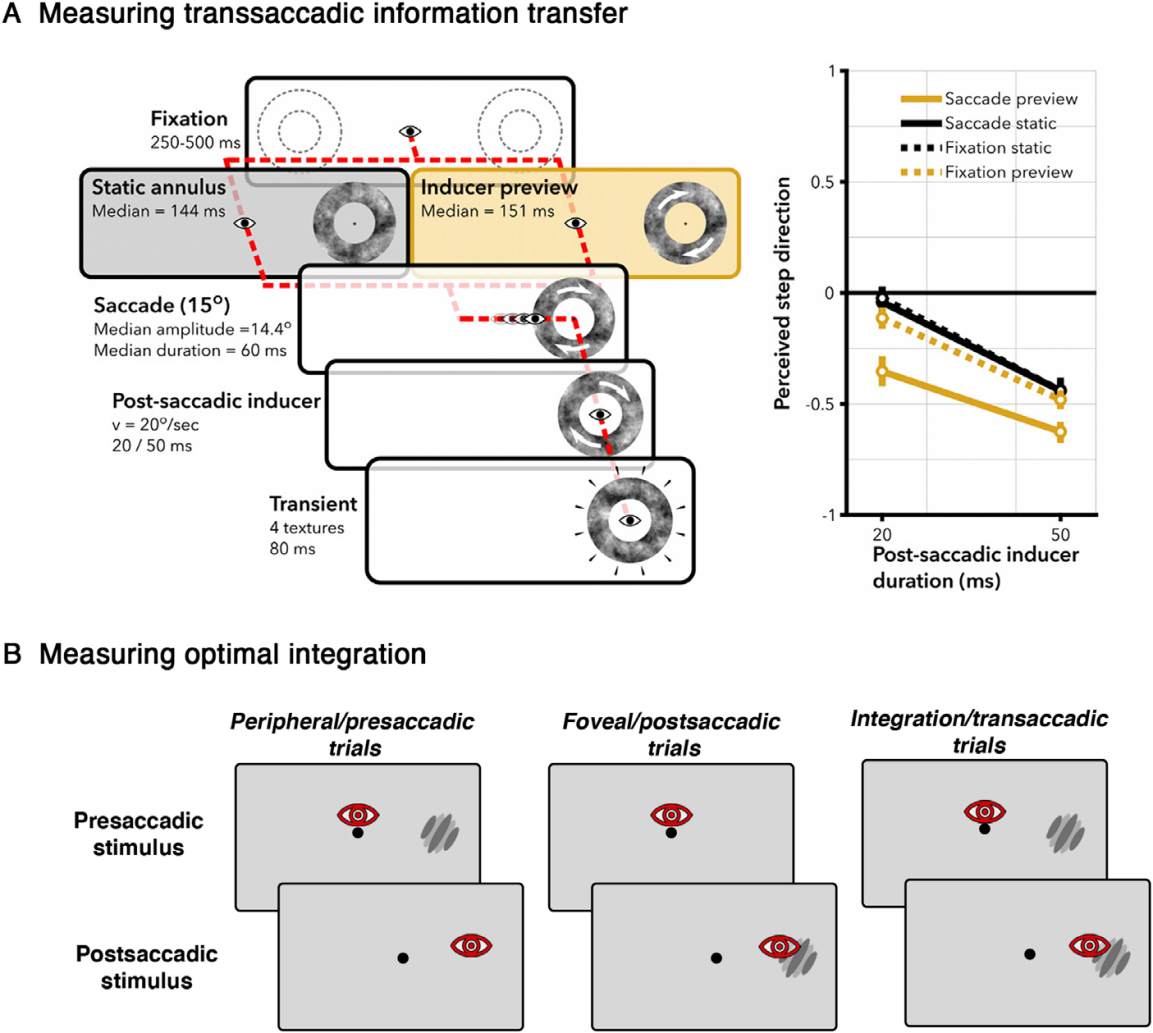
(A) Presaccadic information can affect postsaccadic perception. Fabius et al. (2019) used the High Phi illusion to measure transsaccadic information transfer (Presaccadic information alters postsaccadic perception). The paradigm is shown on the left, and the results are shown on the right. An inducer stimulus presented before the saccade created an illusory jump in spatiotopically matched stimulus texture after the saccade; this jump was greater when a saccade was executed than in a spatially matched fixation condition, or when the presaccadic inducer was static. Figure modified with permission from Fabius et al. (2019). (B) Optimal transsaccadic integration is tested by measuring the reliability of the pre- and postsaccadic percepts alone, and the reliability of the combined transsaccadic percept. If information is integrated, the reliability in the transsaccadic trials should be greater than in either pre- or postsaccadic trials alone.

We have seen that foveal and peripheral information interact to alter perception, both during fixation and during saccades through re-calibration. When peripheral and foveal information is integrated during a saccade, this interaction follows the temporal order of stimuli seen first in the periphery, and then in the fovea after the saccade, such that peripheral information can alter foveal perception. Fabius, Fracasso, and Van der Stigchel (2016); Fabius, Fracasso, Nijboer, and Van der Stigchel (2019) showed that information accumulated in the periphery is directly able to alter foveal postsaccadic perception (see Figure 6A). They utilized the High Phi motion illusion (Wexler, Glennerster, Cavanagh, Ito, & Seno, 2013), in which a rotating “inducer” stimulus is followed by a static “transient” texture: the inducer causes a perceived “jump” of the texture in the opposite direction to the inducer motion. In the studies by Fabius et al. (2016; 2019) the inducer was presented presaccadically, and the transient postsaccadically: Remarkably, this presaccadic inducer lead to a postsaccadic illusory jump, and the strength of this effect was modulated by the presentation duration of the inducer. While this illusory jump was also present when the inducer and transient were presented in the same peripheral spatiotopic location during fixation, the effect was stronger with an intervening saccade. It is interesting to note that this may imply that enacting a saccade may enhance any temporal integration that may occur during fixation (as outlined at the beginning of this section). This transfer of visual features was also observed on a neurophysiological level (Edwards, Paeye, Marque, VanRullen, & Cavanagh., 2017): the influence of presaccadic information could be decoded from electroencephalogram (EEG) signals briefly after saccade onset, and this presaccadic information was immediately used to bias postsaccadic perception (in this case the pre- and postsaccadic stimuli were either faces or houses, producing very distinct signals). In both of these cases, the pre- and postsaccadic signals were deliberately distinct so that the experimenters could measure the effect of peripheral information on foveal perception. In a natural scenario, however, pre- and postsaccadic percepts are less likely to be so different, hence, it may be functionally useful for the visual system to predict foveal perception based on the peripheral view.

#### Feature integration: Averaging

Presaccadic information encoded in memory has long been known to affect postsaccadic performance, for example, providing preview benefits in object naming (Pollatsek, Rayner, & Collins, 1984; Henderson, Pollatsek, & Rayner, 1987) and letter identification (Henderson & Anes, 1994). This preview effect is reminiscent of even earlier work that showed that a peripheral preview facilitates foveal recognition, even without a saccade (see footnote 3, Wagner, 1918). Transsaccadic priming seemed to work on both a more abstract level, for example, an object or category name, or on a more precise level dependent on the features of the objects (Pollatsek et al., 1984), and this research paved the way for the idea that specific object features could be retained in memory across saccades to facilitate postsaccadic perception. Unlike these earlier transsaccadic priming studies, which focussed mainly on how presaccadic information facilitates speeded object identification responses, the newer wave of transsaccadic feature integration studies have a greater focus on how the presaccadic, peripherally viewed percept of a feature directly interacts, or is combined with, the postsaccadic, foveal percept of that same feature. In the previous section, we discussed cases where the effects of the presaccadic stimulus were immediately measurable in the postsaccadic percept (i.e. Fabius et al., 2016). In this section, we will focus on a different subsection of studies into transsaccadic feature integration, where pre- and postsaccadic stimuli are treated as more discrete elements, and where feature information from both are extracted, encoded into memory, and subsequently combined to form a composite, averaged percept (see Figure 5). When this averaging occurs, the postsaccadic percept is biased toward the presaccadic percept. For example, the shape of a presaccadically presented ellipse will result in a bias toward the perceived shape of the postsaccadic ellipse, such that the reported percept is an average of the pre- and postsaccadic shapes (Demeyer, Graef, Wagemans, & Verfaillie, 2010). Similarly, the perceived color of a color patch presented before the saccade will be shifted toward the color of a postsaccadic stimulus presented in the same spatial location (Wittenberg, Bremmer, & Wachtler, 2008). The weighting with which pre- and postsaccadic information is combined is not, however, equal, rather it occurs in proportion to the relative reliability of each stimulus. When the contrast of incongruent pre- and postsaccadic shapes is varied, the accuracy of identifying the postsaccadic shape depends on both the contrast and congruency of pre- and postsaccadic stimuli (Demeyer, Graef, Wagemans, & Verfaillie, 2009); when the level of noise present in the pre- and postsaccadic stimuli is varied, more weight is given to the more reliable signal, so that the perceived color is biased toward the less noisy percept (Oostwoud Wijdenes, Marshall, & Bays, 2015). As with many other interactions, the fovea also naturally dominates this weighting, given the higher reliability of foveal vision: indeed, when the reliabilities of the pre- and postsaccadic percepts are not equated by experimental manipulation, the percept is dominated by the postsaccadic stimulus (Wolf & Schütz, 2015; Schut, Stoep, der, Fabius, & Van der Stigchel, 2018). While in natural vision it is most likely that the reliability of the postsaccadic percept will be higher than the postsaccadic percept, the weighting mechanism demonstrates that the visual system does not simply assume that the foveal percept will have higher reliability, but is able to flexibly change the weighting of the percepts based on the current input.

#### Feature integration: Reliability benefit

The concept of reliability is central to the next theme of integration that will be discussed in the following section: optimal feature integration (see Figure 5). This aspect of integration focuses on the *reliability* of stimulus information across saccades. For every stimulus, the visual system must estimate the true value of that stimulus, for example, if the orientation of a line needs to be judged, the true orientation of the line must be estimated from the sensory input. If the sensory input is noisy, it may be less clear what the true orientation is, and the distribution of *estimated* true orientations may be wider. When we discuss reliability in this context of integration, we refer to the width of the distribution of a sensory estimate, such as the estimate of the true orientation of the line. Reliability, therefore, is calculated as the inverse of the variance of a sensory estimate: the more reliable the sensory estimate is, the less the signal is corrupted by sensory noise. This measure of integration conceptualizes the relationship between peripheral and foveal information as more than just an averaging, biasing, or altering of one percept based on the other, but it rather suggests that combining pre- and postsaccadic information may result in an integrated percept that is more reliable than either individual percept alone. The framework in which feature-integration benefits have been measured is maximum likelihood estimation (MLE) cue combination (e.g. Landy, Maloney, Johnston, & Young, 1995; Yuille & Bülthoff, 1996). This is commonly used in studies of multisensory integration to determine how information from two different modalities can be combined, for example, visual-haptic information (Ernst & Banks, 2002; Gepshtein & Banks, 2003) or visual-auditory information (Alais & Burr, 2004; Bentvelzen, Leung, & Alais, 2009), and it is also used for integration of different signals within the visual modality alone: for example, the combination of texture and motion (Young, Landy, & Maloney, 1993), stereo and motion (Johnston, Cumming, & Landy, 1994), stereo and texture (Knill & Saunders, 2003), or slant (Backus, Banks, van Ee, Crowell, & Crowell, 1999). According to MLE cue combination principles, when two cues are combined, the reliabilities of the individual cues are summed, resulting in a higher integrated reliability. As the disparity between the reliabilities of the two cues becomes larger, the integrated reliability draws closer to the more reliable of the two separate cues.

Two contemporaneous studies used MLE cue combination to show that, as with many other aspects of visual processing, transsaccadic integration also follows these cue combination principles. Wolf and Schütz (2015) and Ganmor, Landy, and Simoncelli (2015) measured orientation discrimination performance for presaccadic (peripheral), postsaccadic (foveal), and transsaccadic (both peripheral and foveal) stimuli (see Figure 6B). They found that performance was better for the integrated percept than for either single percept, and that this performance was near-optimal according to the predictions from the MLE model. The weighting of pre- versus postsaccadic information was also tested by changing the orientation of the postsaccadic stimulus relative to the presaccadic stimulus: foveal information was always slightly over-weighted, even when the contrast of the stimulus was degraded so that the reliabilities of the pre- and postsaccadic stimuli were matched (Ganmor et al., 2015; Wolf & Schütz, 2015; see also Schut et al., 2018). Despite the overweighting of the fovea, information from the periphery is not discarded unless the disparity between the peripheral and foveal percept is extremely high. When this disparity is not high, the result of combining peripheral and foveal information is a percept that is more reliable than could be provided by either source alone. This suggests a functional interaction between peripheral and foveal vision that goes beyond merely reconciling physiological differences to achieve a stable world-percept. The integrated percept that would produce the least absolute violation of the assumption of a stable world, from either source, would simply be the average of the peripheral and foveal percepts.

It should also be noted that one criticism that has been raised about measuring integration in this manner is that when measuring transsaccadic performance, the stimulus is essentially shown for twice the amount of time as the pre- or postsaccadic stimuli alone (see Figure 6B). Melcher and Morrone (2003) suggested the existence of a transsaccadic temporal integration mechanism by showing that motion discrimination thresholds improved when a postsaccadic motion patch was presented in addition to a presaccadic patch, compared to the presaccadic patch being presented alone. This study suggests that information can build up and be integrated with prolonged presentation of feature-congruent stimuli. Under this assumption, any integration benefits could thus be due merely to increased exposure time of the stimulus. Stewart and Schütz (2019a) ruled out this explanation by comparing performance for integration stimuli presented for a certain duration both pre- and postsaccadically, to predictions from a temporal summation model, based on performance for either pre- or postsaccadic stimuli presented for twice that duration. Here, the temporal summation model actually overestimated integration performance, and the observed integration benefits were less than predicted by this model. This demonstrates that the benefit from integrating pre- and postsaccadic information is not the same as merely having longer exposure to the stimulus. We should also note that Melcher and Morrone's (2003) study was itself criticised, with Morris, Liu, Cropper, Forte, Krekelberg, and Mattingley (2010) suggesting the existence of an alternate mechanism whereby repeated exposure of a stimulus reduces decision uncertainty. The Stewart and Schutz (2019a) study suggests, however, that it is unlikely that transsaccadic integration depends on this mechanism as, unlike in the Morris et al. (2010) study, the integration seemed to occur only in spatiotopic reference frame, and integration performance followed a model assuming that the predominant source of noise that limits performance is at the early, encoding, pre-decision stage.

#### What is integrated, and how does integration occur?

While near-optimal integration was first measured for orientation stimuli, Stewart and Schütz (2018b) showed that near-optimal transsaccadic integration could also occur for another low-level stimulus: color. Integration does not seem, however, to be limited to low-level features: near-optimal integration was also found for higher-level numerosity information represented by a dot cloud (Hübner & Schütz, 2017). In this study, integration benefits were seen despite changes to the composition of the numerosity stimuli across the saccade (luminance polarity and arrangement changes), suggesting that the information being integrated were higher-level summary statistics that accounted for the stimulus as a whole, rather than a low-level retention and comparison of details from every dot in the array. The integration of higher-level summary statistics, and benefit from integrating these statistics, would suggest that integration may be a higher-level interaction between peripheral and foveal stimuli, and that the combination can even occur on a more abstract level of stimulus representation. Given this apparent high-level integration, it may follow that some form of higher-level cognitive resource, such as VWM or attention, may be required for integration to occur. Stewart and Schütz (2018b) directly tested this hypothesis by introducing additional memory load into the integration task. Their study showed that optimal integration was impaired with even a single memory item, and this occurred with both orientation and color integration, and with memory items that had features that were both congruent and incongruent with the integration stimuli. Stewart and Schütz (2018a) similarly tested whether integration relies on presaccadic attention by presenting a colored attentional distracter during an orientation integration task: as with the Stewart and Schütz (2018b) study, integration benefits were impaired by the presentation of this distractor. Van der Stigchel, Schut, Fabius, and Van der Stoep (2020) also showed this reliance on attention in transsaccadic perception, using a saccade adaptation paradigm. In their study, when the locus of presaccadic attention was adapted away from the perceptual target along with the saccade endpoint, presaccadic information had less of an influence on the final percept than in an unadapted condition. Taken together, these studies suggest that in order for integration to occur, the stimulus information must be encoded in VWM and then compared/combined. While it is unclear whether the impact of either the attentional distracter (Stewart & Schütz, 2018b) or of the shift in the locus of presaccadic attention (Van der Stigchel et al., 2020) was due to a requirement for attention per se, or whether the diversion of attention also impaired the encoding, or maintenance of the stimuli in VWM (Bays & Husain, 2008; for a review see Ma, Husain, & Bays, 2014), it is certainly possible that the results of these studies may reflect a deeper interplay between attention and VWM. These findings also suggest that integration of peripheral and foveal stimuli may be a rather resource-demanding task, so this again brings into question how functionally useful integration may be for achieving perceptual stability in daily life.

How does this concept of transsaccadic feature integration differ from fusion? Comparing integration to fusion leads to a multilevel hypothesis about how pre- and postsaccadic information may be combined. The combination of pre- and postsaccadic information can be categorized into two separate mechanisms: low-level perceptual fusion, where a low-level presaccadic stimulus representation is retained and fused with the postsaccadic stimulus, and integration, where the pre- and postsaccadic stimuli are retained in visual working memory and compared, and where the integrated percept relies on properties extracted from both of these stimuli (see Figure 5). Although both accounts allow for the combination of pre- and postsaccadic information, the level at which this occurs is different: for fusion, there is a just a low-level spatiotopic overlay of pre- and postsaccadic stimuli, whereas for integration there is a greater requirement that pre- and postsaccadic stimuli are encoded into VWM, so that feature information can then be extracted from each and used to calculate the integrated percept. Studies have identified different forms of transsaccadic memory representations that reflect different levels of processing (for example, fragile, pre-attentive sensory memory, Zerr, Gayet, Mulder, Pinto, Sligte, & Van der Stigchel, 2017; see also Aagten-Murphy & Bays, 2018, for review). These different levels of integration may be accounted for by different levels of transsaccadic memory representations.

Evidence directly supports this distinction between low-level fusion and high-level integration: Paeye et al. (2017) found that fusion also seemed to occur when eye movements were not being made, suggesting that fusion may just be a general extrapolation of temporal integration, rather than being intrinsically tied to saccade execution. This also differentiates fusion from integration, which was only found to occur when a saccade was being executed, but not when the screen was shifted across a stationary fixation in the replication of an eye movement (Ganmor et al., 2015). Additionally, the previously mentioned study looking at the integration of numerosity information (Hübner & Schütz, 2017) rejected the hypothesis that fusion was an underlying mechanism of integration: in a condition where black dots were presented presaccadically and white dots postsaccadically in the same spatial location, a fusion account would cause the overlay of black and white dots to form grey patches, which would have caused a reduction in numerosity estimates for the fused percept. As this reduction was not observed, it is unlikely that fusion occurred in this scenario, but optimal integration was still possible.

The MLE cue-combination model has been used to measure optimal integration, and factors that may cause a departure from optimality, but variations of this model can also be used to infer more about how integration may occur on a neuronal level. Stewart and Schütz (2019a) compared integration performance to both an early and late noise integration model (Jones, 2016). The early noise integration model assumes that sensory noise is incorporated separately into pre- and postsaccadic signals, before integration occurs; the late noise integration model assumes that sensory noise is negligible, and that the predominant source of noise in the process comes later, after integration (this may be more akin to decision or response noise). This study found that in an orientation integration task, integration performance closely matched the early noise model predictions. This indicates that pre- and postsaccadic information may be processed by separate, potentially retinotopic neural populations, each subject to their own, independent sensory noise, and which are integrated in a spatiotopic manner. This also, however, raises an interesting conundrum: as we have discussed in the previous sections, foveal and peripheral processing are intricately connected, and it is unlikely that, in the case of integration, either percept is truly independent from the other.

#### Where in the visual field does integration occur?

Many of the aforementioned studies have focussed on integration of peripheral to foveal information across saccades, which may be the most natural scenario to consider in terms of reconciling pre- and postsaccadic inputs. However, integration may be a mechanism that acts in a more general manner to reconcile presaccadic and postsaccadic peripheral information presented in the same spatiotopic location. Indeed, spatial information about stimuli at locations in the visual field other than the saccade target can be integrated across saccades: Cicchini, Binda, Burr, and Morrone (2013) showed that location information from pre- and postsaccadic bars presented along the saccade path was integrated to affect perceptual mislocalisation, and Prime, Tsotsos, Keith, and Crawford (2007) showed that observers could localize the intersection of pre- and postsaccadically presented bars. Any errors in this localization task could be accounted for by saccade metrics, suggesting that the visual system accounts for oculomotor movement/error when integrating pre- and postsaccadic information. Indeed, there seems to be a certain tolerance for saccadic error when determining whether features will be integrated: Schut et al. (2018) found no difference in transsaccadic color bias as a function of saccade landing distance from the target. There seems to be a fairly large tolerance for information that is integrated around the saccade end point: this is again in direct contrast to the measures of fusion, which require a precise spatial alignment for an overlay to be observed (Paeye et al., 2017). Stewart and Schütz (2019b) further showed that integration benefits at locations across the visual field did not differ from those at the saccade target, although a nonsignificant trend suggested that this benefit may diminish as the saccade moves away from the tested location. Integration may therefore be a more general mechanism used to reconcile pre- and postsaccadic information at task-relevant locations across the entire visual field, and may not be specific to the integration of peripheral and foveal information, although this may be the most common situation in which integration occurs.

### Integration's limits: Causal inference and perceptual stability

Transsaccadic integration is often promoted as being a useful mechanism for maintaining perceptual stability across saccades: this may, prima facie, seem like an obvious connection, however, it has been recently questioned how much of a role transsaccadic integration may actually play in our percept of a stable world (Stewart & Schütz, 2018b; Stewart & Schütz, 2019b). While there seems to be an obvious benefit from the integration of peripheral and foveal information across saccades, the role of integration in perceptual stability may be more nuanced than providing a simple perceptual benefit or averaging, and there may be limitations on where, and when, integration occurs.

The first such limitation is one that was again foreshadowed by Helmholtz (1867, English translation, 1925):

The peculiar ultimate basis, which gives convincing power to all our conscious inductions, is the law of causation. If two natural phenomena have frequently been observed to occur together, such as thunder and lightning, they seem to be regularly connected together, and we infer that there must be a common basis for both of them (Helmholtz & Southall, 1925, p. 29).

Theories of causal inference suggest that for integration to occur, the brain has to establish such a causal relationship between two objects (Körding, Beierholm, Ma, Quartz, Tenenbaum, & Shams, 2007): if two objects are classified as originating from the same source, they are integrated, otherwise they are segregated. In terms of transsaccadic integration, this means that the pre- and postsaccadic information should only be integrated if they are considered to originate from a single object. This can also be framed as the brain having to determine whether the signals from pre- and postsaccadic objects reflect a stable or unstable world. Atsma, Maij, Koppen, Irwin, and Medendorp (2016) implemented this causal inference framework to explain how information about object location is integrated or segregated across saccades. Observers had to localize pre- and postsaccadic objects that were displaced during the saccade: the extent to which an object was perceived as being displaced (saccadic suppression of displacement [SSD], Bridgeman, Hendry, & Stark, 1975) depended on both the magnitude of the displacement and the postsaccadic presentation duration. With larger displacements, the assumption of the objects arising from a single, stable source is broken, and the objects are segregated rather than integrated. Similarly, Tas, Moore, and Hollingworth (2012) suggested that the transsaccadic perception of object stability is dependent on establishing a correspondence between the pre- and postsaccadic objects. This suggests that in order for transsaccadic integration to occur, a causal link must be established between the pre- and postsaccadic stimuli. The requirement for pre- and postsaccadic stimuli to be causally linked seems to be supported by cases where integration of pre- and postsaccadic stimuli did not occur: Demeyer et al. (2010) and Wittenberg et al. (2008) both showed a reduction in bias from the presaccadic percept when there was a blank between the pre- and postsaccadic stimuli. Demeyer et al. (2010) suggested that, in their case, this may have been due to a decay in a fragile, maskable memory trace; however, the introduction of a blank could have also created a disruption to the assumption that the pre- and postsaccadic information belong to the same object and need to be integrated across the saccade. Cicchini et al. (2013) also saw no interaction between stimuli with large orientation differences, suggesting that this causal link may also be broken when there is a large disparity in object features. Interestingly, as we mentioned previously, near-optimal integration was still found despite transsaccadic changes to the composition of numerosity dot clouds (Hübner & Schütz, 2017): it seems that, in this case, the stimulus changes were not enough to break the causal link between the stimuli as a whole. This may suggest that the rules governing integration versus segregation across saccades are flexible and task-dependent. In the case of numerosity judgments, the information being integrated (the number of dots) did not change, therefore, it is likely that within the framework of this particular task, the causal link between the rather abstract feature of interest was not broken.

In addition to their other findings, using the SSD paradigm described above, Atsma et al. (2016) also showed that when the postsaccadic stimulus presentation time is longer, displacement estimates are drawn more toward the more reliable signal that stems from this longer presentation time. In this case, segregation, rather than integration, occurs. Similarly, Zimmermann, Morrone, and Burr (2013) found less SSD with shorter presaccadic stimulus presentation durations. These findings reflect the transsaccadic integration studies, which show that more weight is given to the more reliable signal (Wolf & Schütz, 2015; Ganmor et al., 2015): if one signal is less reliable, the MLE cue-combination model predicts that the integrated percept will reflect the more reliable signal (Ernst & Banks, 2002). Therefore, for integration to be observable, the signals should not only be causally linked, but should not be disproportionate in their reliabilities.

A causal connection between pre- and postsaccadic objects would be completely aligned with the concept of integration as a perceptual stability mechanism: if the objects are considered to have originated from the same source, then they are integrated; otherwise, segregated. The second limitation of integration, however, questions the extent to which integration may actually be a useful mechanism for perceptual stability. This becomes evident when considering the aforementioned findings, that optimal transsaccadic integration can be destroyed by the presentation of a single attentional distractor, or by a single item held in VWM (Stewart & Schütz, 2018a; Stewart & Schütz, 2018b). If integration cannot occur with any additional attention or memory load, then how can it be a useful mechanism to maintain perceptual stability in a visual world cluttered with items that demand attention and memory resources?

As has been previously argued (Stewart & Schütz, 2018b; Stewart & Schütz, 2019b) it seems likely that integration is a specific mechanism that may occur only at attended, task relevant locations, where integration would be important to help alleviate the differences between the attended peripheral and foveal percepts. While peripheral information may be encoded for all other locations, the subsequent processing of this information may be limited due to inattentional blindness (Simons, 2000), or decision complexity limitations (Rosenholtz, 2020), so that there is no awareness of discrepancies in the pre- and postsaccadic percepts at these task-irrelevant locations. There may be no need for integration in this case, as transsaccadic changes in perception may not reach conscious awareness, and therefore would not need to be compensated to maintain a stable world percept.

Given these considerations, we would speculate that integration occurs when an object of interest is selected using peripheral vision, and a saccade is executed in order to scrutinize it with foveal vision. Integration would be needed to reconcile the attended peripheral percept of the object with the subsequent foveal percept. Here, with enhanced acuity and sensitivity at the upcoming saccade location (e.g. Hoffmann & Subramaniam, 1995; Kowler, Anderson, Dosher, & Blaser, 1995; Deubel & Schneider, 1996; Rolfs & Carrasco, 2012), the reliability of foveal and peripheral percepts may also be more equated, so integration of the signals would be consistent with the MLE model, and with the Atsma et al. (2016) integration/segregation reliability model.

In the alternate situation where a person may be idly scanning their surroundings, it may be quite likely that there is no need for peripheral and foveal information to be integrated, and perception may rely on pre- or postsaccadic percepts alone. This explanation reconciles the many studies showing that integration can occur (all of which were tested in circumstances where the integration stimuli were task-relevant and attended), with evidence that suggests that integration can be disrupted with very little attentional or memory load, and is consistent with the large body of studies on change-blindness that suggest limited memory for scene content (Grimes, 1996; Rensink et al., 1997; for reviews, see Simons & Levin, 1997; Rensink, 2002; Simons & Rensink, 2005). This is also in line with the idea that perceptual stability may occur via the allocation of attentional pointers to relevant locations in the visual field (Cavanagh, Hunt, Afraz, & Rolfs, 2010; Mathôt & Theeuwes, 2011).

### Open questions

1. *Is integration automatic?* As we mentioned in the previous section, it is likely that integration occurs at attended, task-relevant locations, and may not be an automatic process that accompanies every saccade. Under what circumstances does integration occur - does there need to be a specific perceptual task to necessitate integration? Further, if, as we have discussed in the previous section, integration may occur in order to intentionally scrutinize an object with more precision, the question arises as to whether all object features are integrated, or just the most prominent feature dimension(s) for the task. Given that multiple features and complex objects can be retained in VWM (Luck & Vogel, 1997; Awh, Barton, & Vogel, 2007), it is possible that all features, or the complex object as a whole are integrated, however, studies thus far have only measured the integration of a single feature at a time. It is also unknown to what extent stimuli in different spatial coordinates may be integrated. Stewart and Schütz (2018a) used a colored distractor to draw the locus of attention away from the integration stimulus whose orientation had to be estimated: what would happen if the distractor was similar to the integration stimulus and also contained orientation information? Integration seems to occur in a spatiotopic reference frame (Stewart & Schütz, 2019b), but if a distractor shares features with the integration stimulus, causal inference might predict that this newly attended stimulus with similar features could be integrated.
2. *Independence of signals.* As we have seen in the previous two sections, peripheral and foveal inputs interact and influence each other, with effects measurable on both the behavioral and neural level. However, the MLE model used to test optimal integration assumes that the two signals being integrated are independent. How do we reconcile these findings of near-optimal integration, despite the violation of this assumption of independence? As the amount of correlation between the signals increases, the benefit derived from integrating these signals should decrease (Jones, 2016). One possible implication is that the interactions between the signals are quite subtle, so that the benefit gained from integration is still quite high. Another is that foveal and peripheral information can interact on numerous processing levels, and the interactions outlined in earlier sections occur on a different level to integration: in this case, the representations being integrated may not be subject to the same correlated noise. Nevertheless, it may be the case that an integration model that accounts for correlated noise (or redundancies between signals; Oruç, Maloney, & Landy, 2003; Jones, 2016) would be more suitable for situations where peripheral and foveal signals interact.
3. *Could integration rely on peripheral-foveal connections?* Following from the previous point, we can speculate that transsaccadic perception may be facilitated by the peripheral-foveal connections outlined above (Foveal-peripheral interactions during fixation). Processing a presaccadic peripheral stimulus in foveal cortex (Williams et al., 2008) could provide a “jump-start” to the processing of that stimulus upon saccade landing. In the context of this section, pre-emptive processing in foveal cortex could (1) contribute to biased postsaccadic/foveal perception based on the presaccadic/peripheral percept (e.g. Fabius et al., 2016; Fabius et al., 2019); (2) account for fusion (e.g. Paeye et al., 2017), if presaccadic/peripheral pattern segments and postsaccadic/foveal pattern segments coincide in foveal cortex; or (3) result in a more reliable estimate of a stimulus if there is an additive effect of the reliabilities of both the foveally processed peripheral information, and the foveally processed foveal information.
4. *How are moving objects integrated?* When an object moves in the periphery, humans typically foveate it with a saccade and then keep it in the fovea using smooth pursuit eye movements (Rashbass, 1961; Dorr, Martinetz, Gegenfurtner, & Barth, 2010; for reviews see Kowler, 2011; Schütz et al., 2011). During the saccade, the object changes its position, and it is unclear whether the visual system is able to integrate pre- and postsaccadic information from different locations. This would require an object-based integration rather than a purely spatiotopic-based integration (Drissi-Daoudi, Öğmen, Herzog, & Cicchini, 2020).
5. *What is the relationship between spatiotopic feature integration and retinotopic after* *effects*? The aforementioned studies showed integration in a strictly spatiotopic reference frame (i.e. information is integrated from the same location in space, but not from the same location on the retina). Critically, this happens even with very short presentation durations of the stimuli and at short saccade latencies. This is different from the transfer of some aftereffects (e.g. the tilt aftereffect) across saccades that can occur in both retinotopic and spatiotopic reference frames (Melcher, 2005; Knapen et al., 2010; Mathôt & Theeuwes, 2013; Nakashima & Sugita, 2017; Zimmermann et al., 2017), and where the build up of a spatiotopic representation takes much more time than typical saccade latencies (Zimmermann, Morrone, Fink, & Burr, 2013; but see Fabius et al., 2019 for a case of rapid spatiotopic updating). This apparent discrepancy in results could be caused by different factors: adaptation and integration might simply tap into different levels of processing that are organized in different reference frames, and that operate on different time scales. Furthermore, even if they occur at the same processing level, multiple reference frames might exist in parallel and might be triggered by task demands (Boussaoud & Bremmer, 1999).

## Future directions

We have already discussed some open questions at the end of each of the previous sections, but there are, of course, additional overarching questions that we want to briefly sketch out here.

### What is the role of foveal feedback signals for transsaccadic perception?

As reviewed above (Foveal-peripheral interactions during fixation), there is converging evidence that foveal feedback signals facilitate the recognition of objects in peripheral vision during fixation. At present, it is unclear whether, and how, foveal feedback signals contribute to transsaccadic re-calibration and integration. Both phenomena involve the joint processing of presaccadic, peripheral, and postsaccadic, foveal information. Neurophysiological studies have concentrated on the (predictive) remapping of receptive fields as a potential mechanism to achieve stability across saccades, although this notion is under debate (for reviews see Bays & Husain, 2007; Zirnsak & Moore, 2014; Bisley, Mirpour, & Alkan, 2020). Irrespective of whether foveal feedback signals rely on the remapping of receptive fields or on another (unknown) neurophysiological mechanism, there is some evidence for a close link between foveal feedback effects and saccades. First, foveal feedback effects do not occur when a saccade is planned to another location (Fan et al., 2016). This suggests that the foveal feedback effect that is typically observed during fixation might be caused by covertly planning a saccade to the peripheral object. However, this is different from transsaccadic integration, which can occur for objects other than the saccade target (Stewart & Schütz, 2019b). Second, the typical time course of foveal feedback signals of about 120 to 250 ms (Fan et al., 2016; Weldon et al., 2016) corresponds well with typical saccade latencies of about 150 to 350 ms (e.g. Munoz, Broughton, Goldring, & Armstrong, 1998). This would suggest that feedback information from presaccadic peripheral vision and feedforward information from postsaccadic foveal vision coincide at the same time in foveal cortex. However, under cognitively demanding conditions, the time course of foveal feedback can also be much slower at about 500 ms (see Figure 2C; Fan et al., 2016). Because it is unlikely that saccade latencies would be delayed to a similar extent, this would mean that feedforward information from postsaccadic foveal vision and feedback information from presaccadic peripheral vision can be temporarily decoupled. It would be interesting to test if this temporal decoupling also eliminates transsaccadic integration, unlike re-calibration that seems to be rather independent of saccade execution and temporal contiguity (Paeye et al., 2018).

### Is re-calibrated information integrated?

As we saw above (Re-calibration of peripheral and foveal vision), the perception of a saccade stimulus feature can be re-calibrated based on transsaccadic changes to that feature. In principle, this might interact with the integration of peripheral and foveal information (Transsaccadic integration of peripheral and foveal information). Is the true physical value of the stimulus integrated, or the re-calibrated percept? The answer to this question depends on the level at which re-calibration occurs. If re-calibration causes a re-tuning of peripheral neurons based on foveal perception, then the answer would be necessarily positive, as the percept would reflect the physiological coding of the stimulus in early visual cortex, so the re-calibrated percept would be integrated. If, however, this re-calibration is more of a higher-level transsaccadic associative learning (Herwig & Schneider, 2014; Weiß et al., 2014; Herwig et al., 2015), then the answer is less clear. As discussed earlier (Open Questions in Re-calibration of peripheral and foveal vision), this associative learning approach would incorporate additional components that could be integrated: the true value of the stimulus, or the percept formed from a learned association. We do not know which of these elements would be integrated, but we can speculate that the answer may depend on the relative reliability of the true value of the signal versus the strength of the learned association: if the reliability of the true peripheral signal is extremely low, then the system may place more weight on prior learned knowledge.

## Conclusions

In this review, we have summarized relevant literature to address three core questions on the interactions between peripheral and foveal vision. Overall, a clear picture of a highly integrated visual system emerges, in which the differences between peripheral and foveal vision are reconciled by a diverse array of mechanisms: during fixation, peripheral vision is enhanced by using foveal feedback signals for object recognition and by extrapolating information from the fovea toward the periphery; a transsaccadic learning mechanism calibrates peripheral and foveal percepts; information from peripheral and foveal vision can be integrated across saccades to optimize the uptake of information. This suggests that despite their large differences in processing and representation, peripheral and foveal vision are more intricately related than commonly assumed.

## Footnotes

1 We want to briefly clarify the terminology here. The rod-free zone, up to a radius of about 0.75 degrees (Curcio et al., 1990), is typically called foveola or fovea centralis, sometimes also simply fovea. The term *fovea* is used with various definitions, resulting in different estimations of size. Intermediate eccentricities between the fovea and the periphery are often called parafovea. Because many perceptual measures continuously decline from the center of the visual field, and because most of the studies and effects we want to discuss in this review are agnostic about the exact delineation of the fovea, we use the term *fovea* colloquially to refer to the center of the visual field, and periphery to refer to the surrounding area.

2 In some exceptions, performance is superior outside of the fovea (e.g. the central performance drop in perceptual segmentation, Kehrer, 1987; Kehrer, 1989 or the absolute sensitivity to light, Crozier & Holway, 1939; Riopelle & Bevan, 1953).

3 An earlier study in the context of reading showed that the presentation of a word in the periphery can facilitate the subsequent recognition of the same word in the fovea, even when the word cannot be identified in the periphery alone (Wagner, 1918). This is reminiscent of the parafoveal preview benefit in reading (for a review see Schotter et al., 2012), and is particularly relevant for the typical temporal sequence of peripheral and foveal stimulation in transsaccadic perception that we discuss in Section 4. Although the direction of this effect is opposite to the foveal-feedback effect, the two effects may be related to each other. Similarly, the foveal-feedback effect may be related to the influence of contextual scene information that disambiguates and speeds up the recognition of embedded objects (for reviews see Barr, 2004; Oliva & Torralba, 2007). Although object and scene information cannot be unequivocally mapped to foveal and peripheral vision, there is some evidence that this effect is present specifically for the interaction of periphery and fovea (e.g. Roux-Sibilon et al., 2019).

4 Notice that the literature on the warping of perceptual space associated with saccade adaptation is very well developed. We do not discuss it here because it does not pertain directly to the relationship between peripheral and foveal perception. We point the reader to the review by Zimmermann and Lappe (2016) for a discussion of the possible mechanisms subtending this phenomenon.

5 We do not have enough space to cover the large body of literature investigating different reference frames (reviewed in Burr & Morrone, 2011; Melcher & Morrone, 2015): these studies suggest that there is a hierarchy of retinotopic and spatiotopic representations, with spatiotopic representations being restricted to higher-level features (e.g. Melcher, 2005) and developing more slowly (e.g. Zimmermann, Morrone, Fink, & Burr, 2013).
